# Network structure shapes consensus dynamics through individual decisions

**DOI:** 10.1073/pnas.2520483123

**Published:** 2026-01-07

**Authors:** J. Hunter Priniski, Bryce Linford, Anna Hirschmann, Sai Krishna Venumuddala, Fred Morstatter, Nancy Rodriguez, P. Jeffrey Brantingham, Hongjing Lu

**Affiliations:** ^a^Department of Psychology, University of California, Los Angeles, CA 90095; ^b^Department of Applied Mathematics, University of Colorado Boulder, Boulder, CO 80309; ^c^Viterbi School of Engineering, Information Sciences Institute, University of Southern California, Marina del Rey, CA 90292; ^d^Department of Anthropology, University of California, Los Angeles, CA 90095; ^e^Department of Statistics, University of California, Los Angeles, CA 90095

**Keywords:** online networks, narratives, group dynamics, digital media, NLP

## Abstract

How do shared narratives emerge in decentralized online networks? Prior research using simplified group coordination tasks (e.g., face-naming) shows network structure shapes group consensus, but the underlying cognitive mechanisms remain unclear. Here, we examine how network structure influences the emergence and semantic content of shared narrative beliefs in experimental online social networks, using natural language processing measures and agent-based modeling. Media content with complex causal structure attenuates network structure effects by encouraging longer exploration of background knowledge. Yet network structure still shapes the narrative content communicated. An embedding-based narrative alignment measure shows that fully connected groups orient their interactions more toward communicating causes of an event, whereas locally connected networks emphasize the event’s effects. A group’s network structure also influences representational and language change in personal narratives: participants in fully connected networks showed the largest increase in causal language in personal narratives written after interaction, which also orient more around the narrative’s causal events.

## Significance

Digital media and online social networks have upended how narratives are constructed and shared, shaping cognition and culture in unexpected ways. Individuals within these networks have increased narrative agency, which enables them to directly contribute to and share evolving stories. Understanding the reflexive processes between individual and networked group narrative dynamics requires new forms of behavioral experimentation and modeling. We conducted a large-scale online social network experiment on narrative interaction, analyzed language dynamics using agent-based modeling, and developed quantitative measures of narrative alignment. Results reveal how network structure interacts with individual decision-making to influence the dynamics and semantic content of shared beliefs, with implications for understanding how narrative information flows through online networks with different neighborhood connections.

Digital media and online networks have transformed how narratives are structured and circulated. Individual and collective sense-making has been transformed by the seemingly ubiquitous production and sharing of hashtags, short-form text, images, and videos (1, 2), with real-world impacts on people’s identities (3, 4), beliefs about religion (5) and science (6), and collective organizing (2, 7–9). Yet it is unclear how individual decision-making interacts with a group’s network structure to shape the dynamics and content of collective narratives. Real-world narrative and decision dynamics are difficult to model (10–12) and social media communication is not amenable to experimental control. Prior social network experiments have demonstrated how network structures shape collective outcomes through naming tasks (13–15). However, the interaction materials in these experiments were designed to study coordination driven by functional communication needs, rather than to examine how individuals’ causal beliefs and personal narratives evolve as networked interactions unfold.

**Fig. 1.**
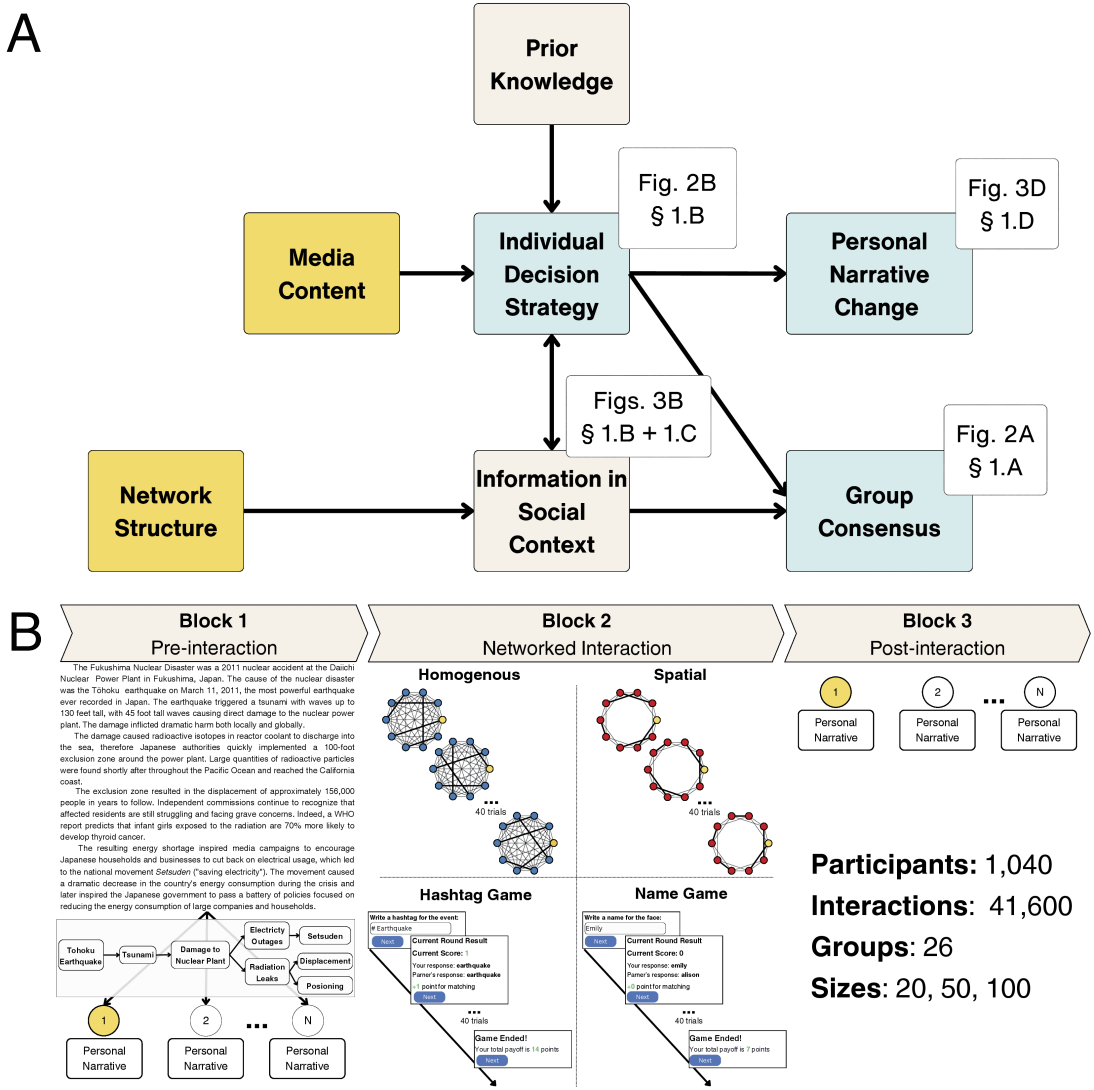
Panel (*A*): Theoretical diagram illustrating the relationships between hypothesized variables. Orange boxes indicate experimental manipulations, tan boxes represent informational inputs influencing decision-making, and teal boxes denote behavioral outcomes analyzed in the results. White boxes show where each set of results or relationships is discussed in the main text, with section numbers indicating where results for each core component are reported. Panel (*B*): Experiment procedure and networked interaction tasks. The experimental design follows three blocks. A single node (in yellow) highlights one participant’s tasks throughout. In the preinteraction block, all participants read the Fukushima nuclear disaster narrative encoding the graphical causal model (not shown to participants), and wrote a tweet-like personal narrative. In the networked interaction block, group communication varied by network structure (homogeneously mixed vs. spatially embedded) and interaction content (narrative via hashtag matching vs. a Name Game control from Centola and Baronchelli, 2015). Participants interacted with network neighbors for 40 trials, receiving one point per matching response; the highest scorer received a financial reward. In the postinteraction block, participants again wrote a personal narrative. See *SI Appendix*, sections S1 and S2 for more information about the experiment environment.

To address this gap, we collected a rich set of narrative interaction data through an online experimental platform spanning multiple network sizes, two distinct network structures, and two types of media content (Fig. 1). Our experimental environment extends long-standing designs using group coordination tasks to measure the effects of varying social network connectivity on convention formation and adoption of shared beliefs (16–21). Participants first read narrative materials detailing a real-world disaster with an inherent causal structure, termed the focal narrative, and were asked to write tweet-like statements about the disaster, termed personal narratives. Subsequently, they were assigned to one of two networked interaction environments. For one group, participants interacted in a Hashtag Game by aiming to generate hashtags that matched those generated by network neighbors and concisely characterized the disaster narrative. For the other group, participants interacted in the Name Game, replicating Centola and Baronchelli’s (2015) face-naming task, grounded in the formal theory of convention formation and illustrating how group-level language conventions emerge from individuals’ functional need to coordinate responses with their network neighbors (13, 22, 23). Both groups were offered identical rewards, incentivizing individuals to match the responses of their network neighbors. The Name Game serves as an effective comparison for narrative coordination in the Hashtag Game because naming conventions, unlike narrative-specific hashtags, are functionally interchangeable and do not require background knowledge about causal event structure.

This study tests how interactions over media with inherent causal structure impact individual decisions and group dynamics, and how network structure impacts the semantic content of narrative interactions. As shown in Fig. 1*A*, we hypothesize that network structure and the causal model evoked by a narrative’s media content jointly influence consensus dynamics through the decisions individuals make to coordinate with network neighbors. Individuals update beliefs and language to coordinate with their social circles by integrating information gathered from social context with their own prior knowledge about the event (24, 25). This real-time integration process impacts both group-level consensus and shifts individual causal understanding about the event over time, even though individuals are only rewarded for successful local coordination with network neighbors without awareness of broader group consensus. This hypothesized framework implies observable effects at both the individual and collective levels. Hence, our analysis examines how the distribution of individual-level decisions shifts over time, alongside the dynamics of consensus formation at the group level. Findings reveal that media content with inherent causal structure encourages individuals to explore their prior knowledge for a longer period of time during networked interaction rather than quickly mirror neighbors’ responses in social context, which attenuates the effect of fully connected network structures on the adoption of shared beliefs.

To better explain the intertwined, bidirectional relationship between individual decisions and how information spreads through social context, we developed a computational network model composed of Context Aware Agents (CAA). The CAA model simulates how individual agents integrate prior knowledge about causal information with social information to coordinate locally. By comparing human and model performance across experimental conditions, we evaluate the model’s ability to capture both the dynamics of human consensus formation and decision strategy change at the individual level. We also compared CAA model simulations with a computational model developed for the Name Game, in which language outputs are functionally interchangeable (26), to highlight the role prior knowledge about narrative information plays in effective coordination. Simulations from the CAA model corroborate experimental results demonstrating that, when agents explore prior knowledge for longer, consensus is more difficult to achieve in fully connected networks.

To examine how the causal content of language data fluctuated during and after networked interactions, we applied two custom-built Natural Language Processing (NLP) pipelines to analyze the semantic content in the networked communications and personal narratives of participants. As described in Fig. 1*A*, network structure influences the array of information individuals encounter in social interaction, which in turn affects their narrative frame; the components of the causal model with which a neighborhood aligns their responses. We developed an embedding-based narrative alignment measure that map hashtags to subcomponents of the narrative’s text, finding that more complete mixing of interactions in fully connected networks orients individual responses toward the causes of the event, while networks with separated neighborhoods reward more divergent local frames with responses clustered around various effects of the event. We also applied a causal language NLP measure (27) to participant’s tweet-like statements to assess how networked interactions influenced the content of personal narratives, finding that participants in fully connected hashtag-matching networks most significantly increased their use of causal language surrounding the disaster’s causes.

## 1. Results

Fig. 1*B* provides an overview of the experiment. We collected 41,600 interactions from 1,040 participants across 26 experimental runs. A total of 989 subjects provided complete interaction data. Participants read a passage describing the 2011 Fukushima nuclear disaster and its effects on local communities and the environment (*SI Appendix*, Table S1). They then composed a tweet-like (preinteraction) personal narrative about the event. Participants were assigned to a network interaction environment to complete either a Hashtag Game, which involved generating hashtags about the disaster narrative during networked interactions, or a Name Game (13) to generate a name for an image of a face (see details in *SI Appendix*, section S2). We also manipulated network structures, including homogeneously mixed (fully connected, N−1 neighbors) and spatially embedded (locally connected, 4 nearest neighbors) networks. Participants were financially incentivized to coordinate responses with network neighbors. Following network interactions, all participants composed another tweet-like (postinteraction) personal narrative.

### 1.1. Narrative Coordination Attenuates the Effect of Fully Connected Network Structures on Consensus.

**Fig. 2.**
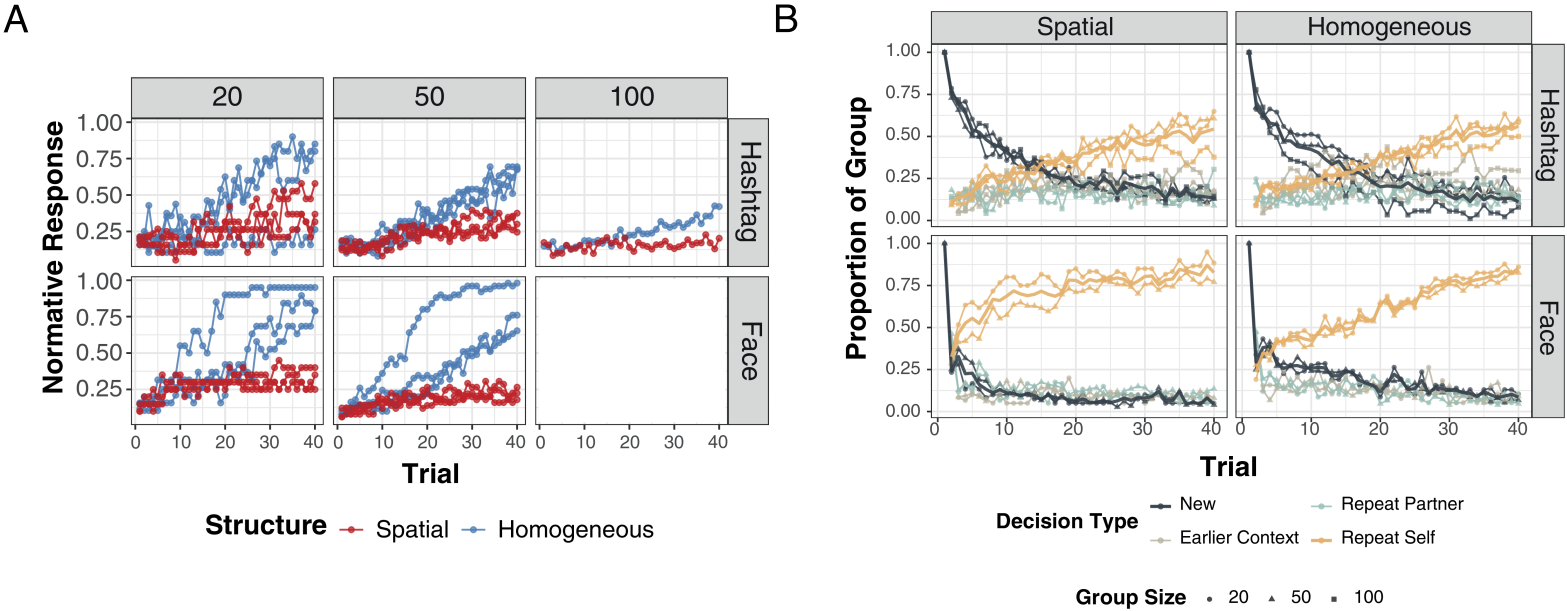
Panel (*A*): Onset of behavioral coherence during networked interaction. Plots show the proportion of each group adopting a dominant response over time, organized by group size (columns) and interaction media content (rows). Each line represents one experimental run, and each point shows the proportion of participants reporting the most common response (which may vary by trial within a run). As shown in the *Top* plots, while groups adopt shared hashtags, they do so more slowly than in the face-naming condition. Changes in response distribution entropy from these runs are shown in *SI Appendix*, Fig. S2. Panel (*B*): Dynamics of individual decisions during networked interaction. Plots show the proportion of each group using one of four decision strategies (sampling new responses, repeating a partner’s last response, repeating one’s own previous response, or resampling from earlier context) over 40 trials, by network structure (spatially embedded vs. homogeneously mixed) and media content (hashtag-matching vs. face-naming). Cases where both the participant and their partner give the same response (i.e., rewarded responses) are coded as repeat self. Proportions reflect averages across runs for each size, structure, and content condition. As shown in the *Top* panels, hashtag-matching groups sample new responses (black lines) for longer, while face-naming groups (*Bottom* panels) adopt a self-consistent strategy (orange lines) more quickly.

We examine the proportion of a group providing the dominant or normative response on each trial as a measure of group-level response convergence (see *SI Appendix*, sections S4 and S5 for details). We fit a Beta-distributed GLM to predict the proportion of a group producing a dominant response as a function of trial number, neighborhood structure, content of network interaction, and interactions between these predictors, while controlling for network size. The data were well fit by a Beta GLM, given that the analysis focused on a dominant response and the response proportions lie between 0 and 1. As shown in Fig. 2*A*, shared responses emerged reliably over time in homogeneously mixed face-naming networks ($\beta_{\mathrm{Trial}}=0.09$, 95% CI [0.09,0.10]), doing so more slowly in homogeneously mixed hashtag-matching networks ($\beta_{Trial:Hashtag}=-0.04$, 95% CI [−0.05,−0.03]), and substantially less so in spatially embedded face-naming networks ($\beta_{Trial:Spatial}=-0.08$, 95% CI [−0.09,−0.07]). These findings are consistent with previous research analyzing group communication over face naming (13). In addition, we found a significant three-way interaction effect between network structure, media content, and the number of trials. The rate at which shared responses emerge across network structures is shaped by the media content ($\beta_{Trial:Spatial:Hashtag}=0.05$, 95% CI [0.04,0.06]). This finding shows that participants interacting over media content with inherent causal relations can reduce the effect of a fully connected network structure on group-level consensus. Specifically, homogeneously mixed networks reached consensus faster than spatially embedded networks for both hashtag-matching and face-naming, but there was less of a divergence in shared responses across network structures in the hashtag-matching condition. We corroborate this finding in *SI Appendix*, section S6 by predicting shifts in the entropy of each group’s full response distribution, which quantifies shifts in the tail of the response distribution beyond the normative response.

### 1.2. Participants Explore New Responses Longer When Coordinating over Media with Causal Structure.

Responses are less interchangeable when they must cohere with a narrative’s causal structure (e.g., hashtags) as opposed to when a shared response is purely conventional, as in the case of assigning arbitrary category names (28) or labels for a face image (13, 22, 23). Hence, we hypothesize that coordinating labels for media content with causal structure increases participants’ exploration of possible responses, thereby increasing the variability of responses in social context and slowing the rate of group coordination, as has been suggested by simulation results of networked group behavior (16). To test this hypothesis in human-based groups, we analyzed how individual-level decision strategies changed over time as a function of network structure and media content. We consider four decision strategies that a participant could adopt while interacting with network neighbors. They could explore the response space by sampling a “new” response that neither a partner nor they themselves had produced on a previous trial. They could copy their partner’s response from the last trial, or repeat their own previous response (“repeat partner” and “repeat self,” respectively). In cases where there is agreement on a previous trial (i.e., repeat partner and repeat self are the same response), we code this as repeat self, as self-consistency is rewarded in these trials. They could also resample a response they remember from earlier interactions (“earlier context”), which could have been self-generated or received in a previous networked interaction trial.

We fit a categorical Bayesian model to predict individual decisions over time across experimental conditions. On average, participants were much less likely to repeat responses from social context in the Hashtag Game than in Name Game ($\beta_{Hashtag:RS}=-1.36$, 95% CI [−1.49,−1.26]; $\beta_{Hashtag:RP}=-1.04$, 95% CI [−1.18,−0.90]); $\beta_{Hashtag:EC}=-0.39$, 95% CI [−0.53,−0.24]). In addition, the onset of different decisions varies across experimental conditions. Fig. 2*B* shows the number of participants exploring new responses (black) and exploiting a response through repeating a past response (orange). Each panel represents a network experiment condition. The number of participants sampling a new hashtag response decreased gradually in the Hashtag Game, but fell immediately for new names in the Name Game.

We hypothesize that prior knowledge about media content and the lack of interchangeability of responses plays a key role in the prolonged exploration and slower convergence seen in the Hashtag Game. As shown in *SI Appendix*, Fig. S6, hashtags generated during network interactions often align with one of the eight discrete causal events described by the narrative. For example, individuals who wrote #Earthquake, #Tsunami, and #Setsuden on a given trial coordinated with their neighbors about 33% of the time. However, the most successful names were Emily (coordination on 65% of trials it was generated), Maddie (60%), and Taylor (60%). Their relative fitness is much higher than the highest coordinating hashtag (#Nuclear), which resulted in coordination around 40% of the time it was generated. Indeed, ten names have higher average coordination rates than the top hashtag. Participants are more willing to adopt names received in social context than hashtags, as there is less background knowledge shaping which responses are viable. Many of the names appear functionally interchangeable in a way that hashtags are not. For example, Mary and Emily are equally viable responses given the lack of causal content communicated by the face stimuli, whereas #Setsuden and #Tsunami isolate different causal relations that are not exchangeable. Therefore, face naming groups quickly reach consensus because responses encountered in social context are readily adopted and do not rely on any underlying causal understanding of media content, in contrast to hashtags about the nuclear disaster.

Interestingly, network structure impacts decision dynamics in the Name Game, but not in the Hashtag Game. As shown in the orange lines on the *Bottom* panels of Fig. 2*B*, spatial (locally connected) face-naming networks show an asymptotic rise in the number of repeated responses, while homogeneous (fully connected) face-naming networks show a linear rise, with both groups reaching about 80% of participants exploiting a response by the end of the experiment (compared to around 50% in hashtag networks). Repeated interactions in smaller neighborhoods can more directly shape individual learning when the interaction media does not require complex causal and situational understanding, as in the case for the spatial face-naming networks. (We discuss how these factors impact the onset of local coordination in *SI Appendix*, section S7 and its implications for what constitutes shared meaning at the group level in *SI Appendix*, section S2.) We observed that network structures do not appear to impact dynamics of the decision strategies employed by individuals in the Hashtag Game. However, these structures could play a role in shaping the content communicated within networked interactions, which we will examine in the next section.

### 1.3. Network Structure Shifts Narrative Alignment of Hashtags Generated During Interactions.

While individual decisions and network structure interact to shape the distribution of responses across a group, these factors may shape the semantic content of responses as well. Numerous studies have shown that causal relations are central to narrative representation (29–32). To compute how hashtag responses aligned with the causal model evoked by the disaster narrative, we developed a narrative alignment measure that leverages sentence-level semantic embeddings to project hashtags onto a lower-dimensional narrative alignment vector. The narrative alignment vector encodes the similarity of a hashtag to different components of the narrative, thus serving as a measure of an individual’s narrative credence at a given point in time.

**Fig. 3.**
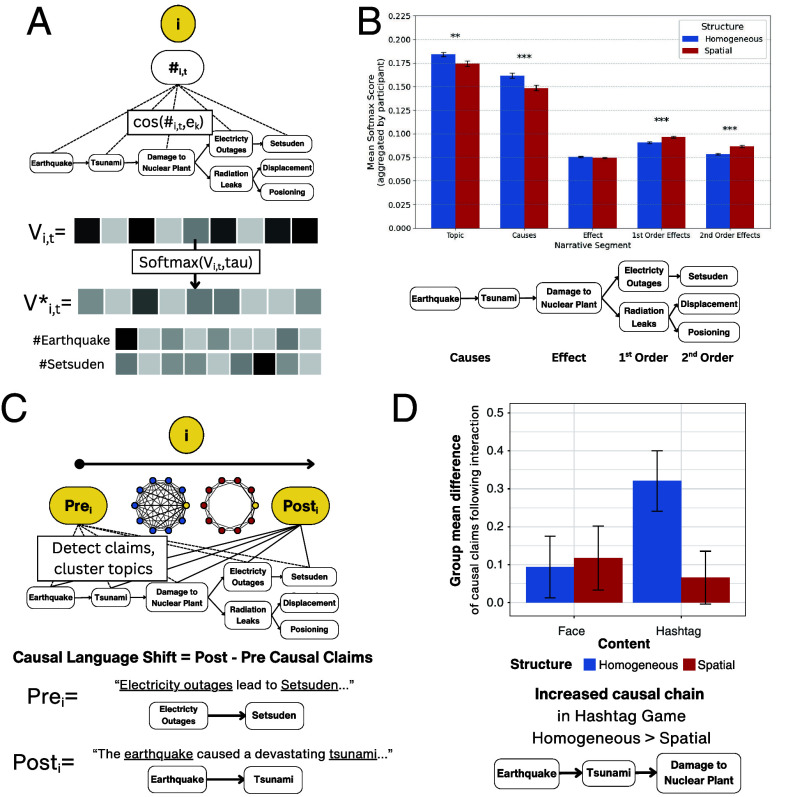
Panel (*A*): The Narrative Alignment Measure aligns hashtags with the causal structure of the disaster narrative. Cosine similarity is computed between each hashtag and the text representing each event in the narrative. The resulting similarity values (one per event) are normalized using a softmax function scaled by tau, producing a vector that sums to one and proxies a narrative credence value. Panel (*B*): Mean narrative alignment score reflects the average alignment vector for each participant (see *SI Appendix* for details). While participants in both network structures generated hashtags aligned with topic sentences and causes of the disaster, homogeneously mixed groups showed greater alignment on these entities. In contrast, spatially embedded groups showed more alignment with the disaster’s effects. Panel (*C*): The causal claims pipeline identifies documents with explicit causal language in personal narratives. If a transformer model detects a causal claim, a causal topic is identified via semantic clustering. Causal language shift is defined as the difference in causal claims before and after interaction. Panel (*D*): Shifts in causal claims following networked interaction. Mean difference scores represent the change in number of causal claims per participant under each interaction condition. Both content conditions shared the same pre- and postinteraction phases (Fig. 1). Participants in homogeneously mixed hashtag networks showed the largest increase in causal language, with most added claims focusing on causes of the disaster.

As described in Fig. 3*A*, each hashtag’s narrative alignment vector is computed by 1) breaking the Fukushima disaster narrative text into causal events based on the underlying causal model; 2) computing embeddings for each hashtag response and for the sentences of each event; 3) computing cosine similarity between each hashtag embedding and each narrative event embedding, yielding a vector of cosine similarities; and 4) computing alignment scores by normalizing the similarity vector with a softmax function such that the values sum to one.

Fig. 3*B* shows that participants in both network structures generated hashtags that aligned more with the narrative topic sentence and causes of the disaster than the effects of the disaster. Importantly, participants in homogeneously mixed networks produced hashtags with significantly higher alignment on both the general topic descriptions of the disaster (t(537)=2.68, P<0.01) and causes of the disaster (t(560)=3.32, P<0.001) compared to those in spatially embedded networks. In spatially embedded networks, participants generated hashtags that were significantly more aligned with first-order effects described in the narrative (i.e., those directly resulting from the disaster event) (t(565)=−4.42, P<0.001) as well as second-order effects (i.e., subsequent effects indirectly resulting from the disaster event) (t(498)=−5.34, P<0.001). There was no significant difference across network structures in hashtag alignment to the damage to the nuclear power plant event (t(568)=0.99, P=0.324).

The asymmetry in narrative alignment of generated hashtags across network structures is likely due to divergences in which narrative information is rewarded and communicated via participants’ social interactions. Social rewards can steer individuals away from background priors during networked interactions (*SI Appendix*, Fig. S5), resulting in smaller neighborhoods coordinating around lower-prior hashtag responses (i.e., shifting away from topic and disaster causes toward effects of the disaster). Participants in homogeneously mixed networks are exposed to responses from a wider array of participants than those in spatially embedded networks (N−1 participants in homogeneously mixed networks compared to 4 neighbors in all spatially embedded networks). Indeed, homogeneously mixed participants are embedded in social contexts with higher entropy (i.e., variation) among partners’ hashtag responses (t=3.35,P<0.001) than participants in spatially embedded networks. Therefore, participants in homogeneously connected networks are more likely to rely on hashtag responses representative of background priors and causes of the disaster event. In contrast, the smaller social neighborhoods and increased repetition of pairwise interactions in spatially embedded networks can steer people away from background knowledge. The lack of across-network connections in spatially embedded networks can further lead individuals to become entrenched in localized narrative frames (i.e., those resulting from coordination rewards rather than background knowledge, which is assumed to be constant across participants). As a result, participants in separable social neighborhoods in spatially embedded networks learn to align hashtags with different features of the focal narrative, including different effects due to increased social reinforcement in smaller neighborhoods. Priors win out quickly in homogeneously mixed networks, where there is more mixing of hashtags (33). To illustrate the semantic content of shared responses emerging across conditions, *SI Appendix*, Figs. S3 and S4 present color maps of full groups’ responses. These figures show how semantic coherence is achieved as agents mix their responses across smaller (N=20) and larger (N=50) groups.

### 1.4. Fully Connected Networks Shift Causal Language of Personal Narratives Toward Causes in Focal Narrative.

Before and after network interaction, participants wrote tweet-like personal narratives about the Fukushima nuclear disaster. We used a causal language analysis pipeline (27) to analyze the narratives generated by participants. The causal language analysis pipeline identifies causal tuples in each document. A document is labeled as having a causal relation if there is a span of tokens belonging to a cause and a span of tokens belonging to an effect within the document. The algorithm then finds cause and effect topics without supervision by clustering the cause-and-effect spans based on their semantic topics (see *SI Appendix*, section S11 for details on the pipeline). The pipeline automatically identified each of the causal events in the disaster narrative in addition to semantically related topics that were not explicitly described in the narrative text (*SI Appendix*, Table S5). We examine how these topic distributions change in personal narratives composed in the pre- to postinteraction phases of our experimental networked environment.

We conducted an independent-sample *t* test on the difference scores (i.e., number of causal claims generated after interaction minus number of causal claims generated before interaction for each participant) across both levels of network structure and media content. As shown in Fig. 3*D*, hashtag-matching, homogeneously mixed networks yielded a significant increase of causal language after networked interactions (t(261)=4.01,P<0.001). Neither hashtag-spatial (t(257)=0.95,P=0.345), nor the face-naming networks had a significant shift (homogeneous difference values, t(201)=1.16,P=0.249); spatial: t(203)=1.40,P=0.164). *SI Appendix*, Fig. S7 shows the distribution of difference scores in each of the network structure and interaction content conditions. To ensure that this effect is robust we additionally fit a Gaussian hurdle model to the distribution of difference scores as a function of network interactions conditions as linear predictors (structure and interaction content) (*SI Appendix*, section S11).

We now narrow our analysis of causal language shifts to the 67% of participants who expressed at least one causal claim identified by the pipeline. We analyzed which specific causal relationships increased and decreased in personal narratives after networked interactions. We computed the subject-level difference scores (postinteraction count minus preinteraction count) for each causal relation for each participant. We then performed one-sample *t* tests coupled with a multiple comparison correction procedure using a False Discovery Rate of 0.05 (*SI Appendix*, Tables S6–S9). For both of the hashtag-matching groups, causal language change centered around the three causal events (“Earthquake,” “Tsunami,” and “Nuclear Disaster”) describing the generative causal chain in the narrative. Furthermore, homogeneously mixed hashtag-matching interactions resulted in significantly more participants in the group eliciting the full causal chain in their personal narratives, that is Earthquake causes Tsunami (P=0.011), and Tsunami causes Nuclear Disaster (P=0.018). The spatially embedded hashtag-matching networks showed a smaller shift toward the initial generative causal chain (P=0.048 for both causal relations). This shift in causal language is concordant with the narrative alignment results for hashtags generated during the networked interaction phase. While both groups were more prone to generate hashtags that signaled the causes of the disaster, homogeneously mixed groups were more likely than spatial groups to generate hashtags that aligned with the causal events. The results differ for the face-naming groups, where the causal chain was not significantly increased in either network condition. These findings suggest that network interactions that produce wider exposure to the causal and semantic content via only hashtag responses have a substantial impact on the causal content referenced in participants’ personal narratives of the event. This network interaction effect on causal language is less pronounced in spatially embedded networks, and nearly absent when groups coordinate around causally irrelevant materials (i.e., naming a face rather than writing hashtags for the narrative).

## 2. Simulating Consensus Dynamics with Networks of Context Aware Agents (CAA)

To model how network structure and decision strategies jointly influence the onset of consensus, we developed a network model of Context Aware Agents (CAA) to simulate the real-time updating of individuals’ decision strategies for integrating their prior knowledge with information gathered from social context during network interactions over the course of the experiment. Based on a parameter α, agents sample responses from prior distributions (background knowledge about possible responses in face or hashtag conditions, see *SI Appendix*, Fig. S5) or from social context (memory trace of interaction history). Background knowledge about media content becomes progressively less important and reliance on social context increases as the number of trials increases. See *SI Appendix*, section S12 for details on the models.

**Fig. 4.**
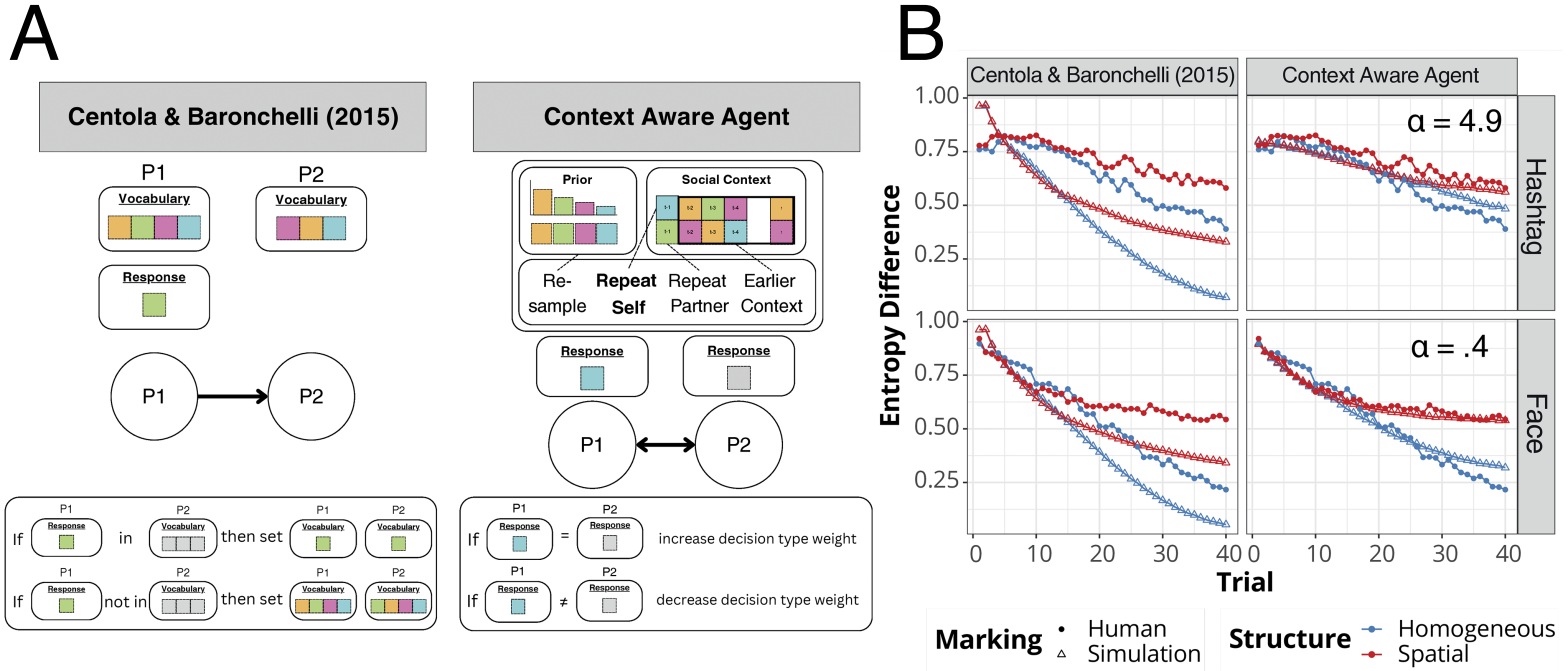
Overview of computational models and simulation results. Panel (*A*) describes the computational model developed for the Name Game (13, 26) and the Context Aware Agent (CAA) model developed in the present paper. The colored squares indicate possible responses in each player’s vocabulary for the interaction tasks (e.g., names in the face-naming task, and hashtags in the hashtag-matching task). The illustrations show how players (P1 and P2) update their vocabulary through the interaction task. Panel (*B*) shows the simulation results of normalized entropy across runs for each content and structure condition against humans from both computational models. α values in the CAA panels measure how much agents sample background priors when generating responses, which attenuates the effect of network structure on group outcomes (the degree of separation between red and blue lines at trial = 40).

We compared the CAA model performance with simulations from the computational model implemented for the Name Game (13, 26). As shown in Fig. 4*A*, the comparison model randomly assigns one individual from each pair to be the speaker and the other to be the hearer. If the speaker’s response is already in the hearer’s vocabulary, each of their vocabularies is updated to contain only that response, otherwise, the speaker’s response is added to the hearer’s vocabulary. The next response of each individual is a selection from their own updated vocabulary. In comparison to the CAA model, the comparison model does not take prior knowledge into account, and thus does not weigh between social context and background information.

Fig. 4*B* shows the entropy results for all four experimental conditions, where we compared experimental results (open points) with model predictions (triangular points). As shown in the *Left* subplots of Fig. 4*B*, we find that the comparison model without considering prior background knowledge consistently overestimates the rate of entropy decrease and does not change for either form of interaction media. These results stem from the lack of exploration of background knowledge in this model; it only takes advantage of an agent’s social context and is a greedier social learning algorithm than the CAA model. The CAA model provides a better fit in all four experimental conditions as shown in the *Right* subplots of Fig. 4*B*. We also find that the CAA model is robust to parameter variation. For example, the performance remains highly similar whether all agents use the same parameter value of α or individual agents draw values from a distribution (*SI Appendix*, section S12).

## 3. Discussion

Demonstrating how individual decisions interact with neighborhood structure to shape the flow of narrative information in social networks is critical for understanding how decentralized groups can reach consensus over complex information. We replicated long-standing empirical findings that information can readily spread across homogeneously mixed (fully connected) networks, which facilitates shared language and beliefs about narrative materials. However, when coordinating labels based on narrative stimuli with inherent causal structure, participants explore possible responses for longer, leading to increased uncertainty in social context and a slower onset of shared beliefs. Well-demonstrated network effects on collective beliefs are therefore attenuated by the causal understanding involved in individual decision-making. Network structure still impacts the content of narrative interactions, however. Complete mixing during interactions in fully connected networks results in individuals orienting their responses toward the causes of an event. Local mixing in spatially embedded networks reinforces individual responses toward disparate narrative effects. Furthermore, groups in the homogeneously mixed hashtag-matching condition significantly increased the amount of causal relations in personal narratives after networked interactions, explicitly mentioning the causes of the disaster event. This shift in personal narratives suggests that exposure to causal information extracted from narrative interactions during network interaction (here via hashtags) has the potential to direct attention to specific features in long-form narratives and shift language used to describe those narratives.

These findings suggest that interventions to foster narrative consensus should look beyond network rewiring. Future experiments might look at the interaction between network structure and the causal framing of a narrative’s situation to manipulate the communicative context of a networked group: shifting how individuals frame a narrative prior to networked communications in order to establish the group’s narrative frame from which communication and social learning is carried out. Experiments on individuals show that metaphors can frame complex social phenomena and set narrative frames that impact individual’s beliefs and policy decision-making (34). Network extensions of these framing experiments can test how certain frames can more effectively impact group consensus dynamics. A limitation of our study is that the NLP models only focus on how language data aligns with the causal structure of the narrative stimuli. Network experiments on narrative frames will require NLP advances to better probe alignment with a narrative’s complete situation model, which includes elements of agency, time, and space in addition to causation (29, 31, 32). Future NLP models should parse a wider array of semantic relations to model shifts in situation models. Such models can more precisely measure how narrative frames vary across a group and change over the course of interactions with network neighbors.

In this experiment, coordinating hashtags about a focal narrative serves as a proxy for how individuals use hashtags to coreference documents and enable broader narrative collaboration across real-world online social networks (9, 35, 36). Focal hashtags are key for mapping personal narratives to a shared discussion and allow for collective organizing and discussion through hashtagging personal narratives. While direct incentives for using an effective focal hashtag on social networks in the wild do not exist, our experiment platform approximates the utility of gaining traction (i.e., virality) through coordination rewards. Future experiments will use narrative materials that probe political reasoning and more emotional valences, including power, to test how different narratives can extend the experiment platform to study narrative dynamics akin to those in online networks found in the wild.

## 4. Materials and Methods

This section includes material first presented in JHP’s doctoral dissertation (37), and in conference articles presented at the Cognitive Science Society (38, 39).

### 4.1. Preregistration.

We preregistered the experimental design, key hypotheses, and statistical analysis framework on the Open Science Framework at https://osf.io/598dt?mode=revisionId=view_only=. All code and anonymized data for the presented results and software for replicating the experiments can be found at the following GitHub repository https://github.com/jpriniski/NetCom.

#### 4.1.1. Participants.

We sampled a total of N=1,040 participants from the Prolific and UCLA SONA subject pools, and placed them into one of 26 experimental runs. A total of 989 subjects provided complete data. Experimental runs vary according to three factors: the *size* of a network (N=20,50,100), its connectivity structure (homogeneously mixed/fully connected; spatially embedded/ring-like), and the content of interaction (hashtag; face-name) (total nodes N=1,040). We collected a total of twelve experimental runs for face interaction (three runs for each network structure of sizes N=20 and N=50), and fourteen experimental runs for hashtag interaction (three runs for each network structure of sizes N=20 and N=50, and a single run of each network structure for N=100). Participants N=20 and N=50 conditions were sampled using Prolific. For the N=100 condition, we recruited undergraduates in the Department of Psychology at UCLA through SONA subject pools. We posted initial recruitment surveys a week prior to each run in SONA and a few hours prior to each run in Prolific. Participants who received the most points at the end of the experiment received an additional $10 bonus. All experiments and analyses were approved by the UCLA institutional review board under submission IRB-22-1184. All participants provided informed consent prior to participation.

#### 4.1.2. Materials.

Across all network conditions, participants first read a four-paragraph narrative description of the 2011 Fukushima nuclear disaster prior to interaction in a network. The narrative explains how a large earthquake triggered a tsunami that caused damage to a nuclear reactor and resulted in radiation leaks, population displacement, and an energy-saving movement “Setsuden.” We selected this narrative based on a pilot study demonstrating that it resulted in the most diverse set of hashtags within a set of tested narratives related to natural and financial disasters. This is likely because the narrative describes a rich set of causal relations (a generative causal chain producing a branching common cause sequence) and included both negative (e.g., displacement, poisoning) and positive effects (e.g., energy saving movement). *SI Appendix*, Fig. S1 illustrates the causal structure of the Fukushima disaster narrative in *SI Appendix*, Table S1.

#### 4.1.3. Experimental design.

We used the open-source framework oTree written in Python (40), and hosted experiments on a Linux server. Participants joined the experiment through a Qualtrics survey that directed participants to the network experiment.

Our social network experiment proceeded in three steps. First, we randomly assigned each participant as a player in a network that defined who may interact with whom on a given trial. Second, we assigned interactions between individual participants on each trial. Third, we rewarded participants based on the outcome of their interactions. We can specify this process using graph theory notation. The first step is to initialize a fixed graph G(N,E), defined by a set N nodes representing individual participants connected through an edge set E. We discuss below the specific graph structures used. The second step iterates over T trials. On a given trial t∈T, connection (edge) configurations follow mixing participants randomly within a participant’s neighborhood. The third step is to identify and reward coordinated behavior. If the response from participant $n_{i}$ on trial t is $r_{i}^{t}$, then participants $n_{i}$ and $n_{j}$ coordinate if $r_{i}^{t}=r_{j}^{t}$.

#### 4.1.4. Procedure.

The experiment consisted of three blocks: a preinteraction block, a networked interaction block, and a postinteraction block, as shown in Fig. 1. In the **preinteraction block**, participants read a four-paragraph narrative describing the Fukushima nuclear disaster, and then were asked to write a “tweet” (within a 140-character limit) and ten hashtags characterizing the events described in the narrative.

**Fig. 5.**
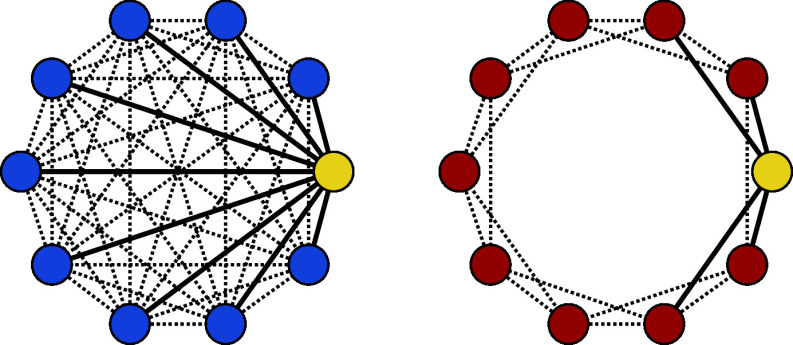
Two network structures tested in this experiment. Homogeneously mixed (*Left*) and spatially embedded (*Right*) networks with N=10 nodes. Edges drawn with a solid line represent the neighborhoods for a hypothetical node 1 (colored yellow) in both networks. As a network’s size grows, the diameter of spatial networks grow whereas homogeneous networks maintain a diameter of 1.

In the **network interaction block**, participants joined a network experiment with real-time interaction via an online platform using the Python framework oTree (40). Participants were assigned to one of six experimental conditions based on the size of the network (N=20; 50; 100) and network structure (spatially embedded and homogeneously mixed; see Fig. 5). Regardless of network size, nodes in spatial networks have a consistent neighborhood size k=4, meaning each participant would interact with only four other participants during the entire experiment. Neighborhood size in homogeneous networks is N−1, as each participant can interact with any of the remaining participants. A consequence is that the network diameter (i.e., the largest geodesic distance in the connected network) was consistently 1 in all tested homogeneous networks, but grows as a function of size in spatial networks. Previous research showed that both features of network topology (i.e., neighborhood size and network diameter) uniquely influence the emergence of shared behavior in online networks (41).

The networked interaction block consisted of 40 trials, in which participants interacted with their partners based on the edge structure in the assigned network. In the Hashtag Game, on each trial, participants were instructed to write a single hashtag describing the narrative they read in the preinteraction block. After participants submitted their hashtag response, they were then presented with a new page showing their own hashtag response, their partner’s hashtag response, whether they received a point for matching responses with their partner, and their cumulative reward points. In the Name Game, on each trial, participants were shown an image of a woman’s face (same image across all trials) and asked to write a name that describes the face. After participants submitted their name response, they were presented with a new page showing their own name and their matched neighbor’s name, whether they received a point for matching, and their cumulative rewards.

Following the networked interaction block, participants entered a **postinteraction block** in which they wrote one more “tweet” for the same narrative and another ten hashtags describing the Fukushima nuclear disaster before providing demographic information. One consequence of these two network structures is not only who is connected to who, but also the amount of repeated interactions a participant has with their neighborhood. The expected number of times a participant interacts with their full set of neighbors across an experiment is $\frac{T}{N-1}$. This means, in the fully connected condition (i.e., homogeneously mixed), participants are expected to interact with their full set of neighbors 2 times when N=20, 81.6% of their neighbors when N=50, and 40.4% of their neighbors when N=100. Meanwhile, regardless of the size of spatially embedded network, participants will interact with their full set of neighbors a total of 10 times across 40 trials. These conditions allow us to contrast the effect of neighborhood size relative to ties across the network, and to determine the impact of repeated interactions between pairs of partners to produce dominant behaviors (e.g., participants in the network responding in a consistent manner).

## Supplementary Material

Appendix 01 (PDF)

## Data Availability

Anonymized experimental data and code have been deposited on GitHub at https://github.com/jpriniski/NetCom (42).
