# Supplementary material for: Network structure shapes consensus dynamics through individual decisions
Source: Proc Natl Acad Sci U S A. 2026 Jan 7;123(2):e2520483123. doi: 10.1073/pnas.2520483123 (PMC12799169; doi:10.1073/pnas.2520483123)
Supplement: Supplementary file 1 — Appendix 01 (PDF) [file pnas.2520483123.sapp.pdf]

Supporting Information for

# Network structure shapes consensus dynamics through individual decisions

**Priniski, et al.**

**Corresponding Author: J. Hunter Priniski**
**E-mail: priniski@ucla.edu**

## S1. Group consensus in homogeneously-mixed and spatially-embedded networks

Researchers have used relatively simple decision-making tasks to study the effects of varying a social network's connectivity on the adoption of shared beliefs in networked groups (1, 2). For example, Centola and Baronchelli (2015) introduced the *Name Game* by asking participants to coordinate with network neighbors on a face-naming task (3). The Name Game extends formal theories of convention formation (4, 5) by experimentally measuring how groups converge to a shared convention through local coordination of labeling a stimulus such as a visual object (6) or a face image (3). Participants learn to exploit word labels encountered in social context to collectively assign an identical referent to a stimulus. Beyond serving as a group referent to the object, the content of a group's agreed on label is otherwise arbitrary.

Consistent with Centola and Baronchelli's (2015) findings, other empirical and modeling studies demonstrate that group consensus can result from individual-level coordination incentives among interacting network neighbors. The dynamics of consensus formation depend in part on how information can move through the network (7–11). Specifically, in *homogeneously-mixed* or fully-connected networks, where each participant can potentially interact and coordinate language with any other member of the network, interactions tend to converge rapidly to consensus. In contrast, in *spatially-embedded networks* or locally-connected networks, where each participant is linked to only a handful of close-by neighbors, such rapid convergence does not occur; instead, separable clusters emerge within local neighborhoods (11, 12).

## S2. Differences between the Name Game and Hashtag Game

We discuss in more detail the differences between the Name Game and the Hashtag Game as coordination tasks for studying group behavior and consensus formation. The first difference concerns the interchangeability of candidate responses. The Name Game extends Wittgenstein's model of language and meaning (4) and David Lewis' formalizations of social convention formation (5). In these frameworks, the word labels that agents choose for referents are largely interchangeable as long as they are functional. For example, it does not matter whether a worker calls an object a "brick" a "slab" or a "rock," so long as there is agreement on which term refers to that object. Agreeing on word labels allows individuals to complete a task together. The Name Game operationalizes this process by requiring participants to converge on a shared label for a face image, where candidate names are largely interchangeable. For example, labels like "Mary" and "Emily" serve the same function for naming a female image. As a result, an individual's prior knowledge of possible face names is more uniformly distributed before networked communication than in the Hashtag Game.

As shown in Figure S5, we measure individual priors by computing the distribution of first trial responses across experimental conditions. For face names, the more uniform-like shape of the prior distributions suggests that many conventional labels are acceptable for naming a face image, whereas the highly skewed prior distributions in hashtag responses indicate that some hashtags provide a better fit than others for characterizing the disaster narrative. This difference indicates that hashtags are less interchangeable: they are not arbitrary markers of a narrative's causal content. Consequently, hashtag coordination depends not only on the social learning processes underlying convention formation, but also on cognitive processes that assess how well a candidate response aligns with (a) the causal content of a narrative being interpreted (i.e., a focal narrative, see below), (b) the individual's personal narrative (i.e., their mental model) of the event, and (c) the beliefs inferred from the information generated and communicated by other agents in their social network. Face-name coordination, on the other hand, relies solely on social learning processes for matching utterances with other agents, and is a less cognitively demanding interaction task that does not require constructing or maintaining a mental model.

The second distinction concerns the function of hashtags in real-world social networks which could impact a participant's responses in a controlled Hashtag Game. We expand on differences between the Hashtag Game and real-world hashtag use in the next supplementary section, but discuss how real-world hashtag generation may impact responses in our experiment here. When people use hashtags to tag their content in relation to broader conversation, they use a mixture of focal or individualistic hashtags (13). Focal hashtags act as parsimonious representations of events that enable broader narrative collaboration. Focal hashtags require coordination across network agents in order to match personal narratives across documents (14). Individualistic hashtags allow agents to add a personalized label to their documents to express their self-identity in online spaces (13), which can increase the semantic variability of hashtag distributions. In practice, social media users can append multiple hashtags, which generally include a focal hashtag followed by individualistic hashtags (13). Our Hashtag Game abstracts this process and focuses on the generation and coordination of focal hashtags by requiring a single hashtag per trial. While narrower than real-world practice, this setup captures the pressure toward convergence characteristic of large-scale discourse, and allows for us to use hashtag generation as a lens on people's dynamic causal reasoning about an event. The financial incentive in the hashtag game parallels implicit online incentives, where users gain visibility and broader narrative collaborations by aligning

focal hashtags with others. Thus, the ecological validity of our design lies in modeling convergence dynamics, not reproducing the full ecology of concatenated hashtags.

## S3. Distinguishing between narrative constructs and hashtag generation in real-world networks and experimental social networks

Here we distinguish between four narrative constructs described in this study, and their relationship to group dynamics and hashtag generation in social media and in experimental social networks: focal narratives, personal narratives, collective narratives, and narrative interactions.

*Focal narratives.* A focal narrative is the shared description of an event that participants read to anchor their reasoning and language. Based on the literature on situation models from cognitive psychology (15–18), our working notion of a focal narrative is a text-based passage that describes a causal model, or causal sequence of events, characterizing a situational occurrence. Psychology experiments were designed to measure the causal, temporal, and character information individual participants encoded in memory after reading a focal narrative, termed a *situation model* in the literature. An individual reasoner constructs a situation model, which encodes their beliefs and memory of information, having read a focal narrative. Our network experiment design builds on this approach to measure how the structure of network communications not only impacts an individual's beliefs and language about a focal narrative, but how communications shape the distributional properties of a networked group's interpretation of a focal narrative (i.e., a collective narrative, as described in more detail below). In our study, the Fukushima nuclear disaster narrative – which all participants read prior to networked interaction – served as a focal narrative. The narrative materials provided causal structure and event information that participants drew upon to write personal narratives as tweet-like messages and hashtags during network interaction. The focal narrative ensures that coordination is not arbitrary but that communications are grounded in a shared space of causal information. On social media, a focal narrative can consist of a news story describing an event or social media content that users respond to in comments or share with others.

*Personal narratives.* A personal narrative is an individual-level situation model constructed from reading materials that describe a focal narrative. A personal narrative encodes an individual's beliefs and causal knowledge extracted from a focal narrative's situation model, and is expressed through language. While the terms situation model and personal narrative can be used interchangeably, we use the term personal narrative because it emphasizes the role of language in communicating narrative information with others. While a personal narrative can be represented in language through short-formatted textual messages, the content of an individual's personal narrative concerns their beliefs about the world, such as their personal beliefs or causal representation of a situation described by a focal narrative. Our analysis of personal narratives in the experiment is therefore concerned with aligning the semantic content of personal narratives to the causal contents of a focal narrative that individuals read about the situation. In our experiment, participants expressed personal narratives in tweet-like texts written before and after network interaction. To model the content of their personal narratives, we applied causal language modeling to *infer* the causal beliefs of individuals from the written text documents that they provided. Analyses of these texts allow us to measure how individual causal representations, the core semantic content of an individual's personal narrative, relate to the content of a focal narrative; in addition to how that causal content of communication data shifts through social learning via interactions with others in one's social network. On social media platforms, users convey personal narratives through posts. Platform algorithms distribute posts to users who are likely to identify with or respond to its narrative elements.

*Collective narratives.* A collective narrative is the aggregation of personal narratives across a group. Here, we consider a collective narrative to be a distributional property of a networked group, which encodes information about the aggregation of personal narratives, including beliefs and causal language of individual nodes. A collective narrative is a latent representation of a group's understanding of an event sequence, just as a personal narrative is a latent representation of an individual's understanding of this event sequence. The data representations of personal narrative shift in Figure 4D in the main text show how a group's collective narrative can shift following networked interactions. In social media, collective narratives emerge when personal narratives coalesce through repeated sharing, reinforcement, and adaptation. Hashtags are frequently used to co-reference personal narratives across a social media platform to form a collective narrative.

*Narrative interactions.* While the previous three narrative constructs are psychological constructs that require inference techniques to represent and model, narrative interactions are a behavioral output that is directly observable in a network experiment. Narrative interactions refer to the behavioral processes by which individuals coordinate and update their understanding of events through communication. Hashtag matching in our experiment is one such form of narrative interaction designed to model how individuals integrate background knowledge about the event described in a focal narrative with the content of narrative interactions of their neighbors. Through social incentives, groups can learn to align their personal narratives *through* narrative interactions, including hashtag matching. As we demonstrate in our study, narrative coordination can induce representational change at the level of individual personal narratives about the event (i.e., as shown in causal language change and how network structure impacts the narrative content of hashtags proposals). While narrative interactions are behavioral outputs that are directly observable, novel statistical methods are still needed to map their content to situational content embedded in focal, personal, and collective narratives. To this end, we developed the narrative alignment measure, string matching for narrative coordination of hashtags, and entropy measures to capture the distributional properties of a group's narrative interactions. Narrative interaction on real-world social media platforms takes more forms, including sharing or liking a message, writing a hashtag and a post to express beliefs about an event, or performing a dance in a TikTok video.

Now that we have described the four key narrative constructs used in our study, we can discuss more clearly how narrative

processes in our experiment vary from those occurring in real-world social networks. In our experiment, we control discussions to be about a single narrative (see Table S1) and elicit a single type of narrative interaction from participants, here hashtags. Hashtags are a distinctive marker of narrative interaction on social media. They function as concise representations of complex narratives (19–21), and connect spatially disorganized groups according to the content of their shared narratives and goals, thus constituting a potent force for online activism (14, 22). Across an online network, hashtags serve as topic labels for generated content, which assist online platforms with the algorithmic categorization, curation, and dissemination of dynamic social media discourse (23) (e.g., by mapping discrete units of online content shared over time to a single event or discussion) (14). At an individual level, hashtags allow users to signal personal contributions to broader narratives emerging from interactions in an online community.

Based on real-world social media networks, previous research on hashtags has primarily focused on understanding their linguistic and semantic content (13) and modeling the dynamics of their adoption and online spread. For example, mathematical models suggest that dominant (i.e., widely shared) hashtags emerge as a result of a preferential attachment mechanism that increases the popularity of early popular hashtags over time. While many hashtags initially compete for popularity, only a small set of hashtags persist to allow for broader narrative collaboration across the network (24, 25). Rather than studying hashtags as a mechanism to spread narratives in real-world social networks with high information competition, we used hashtag generation as an experimental behavior that participants would be familiar with from real-world networked interaction, but could also be used to probe their causal and semantic reasoning/interactions. Through the narrative alignment measure, we can align hashtags to the causal contents of the focal narrative, which provides a concise, quantitative narrative representation that we could model throughout the course of an experimental run. Specifically, in our experiments we use hashtags as a way to study participant's mental model of the narrative content.

## S4. Computing collective outcomes

When analyzing collective outcomes in our networked groups, we examined two measures of group-level coherence: (1) the proportion of a group reporting a shared, or *normative* response, which we model as a Beta Distribution, and (2) the entropy of the full response distribution of a group (26). When assessing how network structures impact behavioral dynamics, researchers have generally predicted the proportion of a network producing a dominantly shared behavior at a given time (3, 10, 11). Shared normative responses represent the agreement, or consensus, of a group.

Following previous literature (3), we encode a normative response by dividing the number of respondents in a group who produced the most popular response on a given trial by the group size. The response could change from trial to trial. We use a beta-distributed generalized linear model (GLM) to model normative responses because beta distributions are well-suited for predicting proportion values within the range of 0 and 1. Beta distributions are flexible through their parameters to model many kinds of distributional curves. The beta-distributed GLM is known for problems in dealing with the boundary situations when the response proportion is 0 or 1. However, these boundary situations (0 and 1) did not arise in our data. We do not have any zeroes because the analysis aimed to predict the proportion of the top hashtag on a given trial, which is always greater than 0, and no group ever reached 100% (complete consensus).

A shortcoming of predicting proportion values is that it predicts a value of a single hashtag response. This approach does not capture the variability of other alternative non-dominant responses. The full set of responses from a group compose a distribution of behaviors that can be heavy-tailed, multi-modal, or highly skewed — key distributional features not captured by simply predicting the modal response. Hence, we also analyzed the results based on the entropy of the response distribution of a group (using the R package *entropy*). The entropy metric provides a concise measure of response variability across the entire group: lower entropy indicates more similar or coherent responses in the group, while higher entropy suggests greater diversity or variation in responses (26, 27). We computed entropy using the standard Shannon entropy formula:

$$H = -\sum_i p_i \log p_i,$$

where $p_i$ is the proportion of responses in the $i$th category.

## S5. Analytic framework for statistical modeling of group dynamics

We fit Bayesian generalized linear models (GLM) to predict how the two network structures (spatially-embedded vs. homogeneously-mixed structure) and media content (hashtag response vs face-naming) support the emergence of group coherence. We fit separate models to (1) predict the proportion of a group producing a dominant response, and (2) the entropy of the group's full response distribution. We also fit a Bayesian GLM to predict the rate of coordination between participants, and how participants sampled decisions over the course of networked interactions. We assume that the proportion of participants who produced the dominant hashtag on trial $t$ follows a Beta distribution, a commonly used distribution to predict proportion data (28); we used uninformative priors (i.e., $\mathcal{N}(0, 10)$) for regression coefficients. Specifically, the GLM model predicted the coherence value as a function of trial number (i.e., $Trial$) interacted with network structure ($Spatial$ vs. $Homogeneous$) and content (hashtag-matching vs face-naming), while controlling for network *size*. If other predictors are incorporated for a specific model, we specify those below.

The Fukushima Nuclear Disaster was a 2011 nuclear accident at the Daichi Nuclear Power Plant in Fukushima, Japan. The cause of the nuclear disaster was the Tōhoku earthquake on March 11, 2011, the most powerful earthquake ever recorded in Japan. The earthquake triggered a tsunami with waves up to 130 feet tall, with 45 foot tall waves causing direct damage to the nuclear power plant. The damage inflicted dramatic harm both locally and globally.

The damage caused radioactive isotopes in reactor coolant to discharge into the sea, therefore Japanese authorities quickly implemented a 100-foot exclusion zone around the power plant. Large quantities of radioactive particles were found shortly after throughout the Pacific Ocean and reached the California coast.

The exclusion zone resulted in the displacement of approximately 156,000 people in years to follow. Independent commissions continue to recognize that affected residents are still struggling and facing grave concerns. Indeed, a WHO report predicts that infant girls exposed to the radiation are 70% more likely to develop thyroid cancer.

The resulting energy shortage inspired media campaigns to encourage Japanese households and businesses to cut back on electrical usage, which led to the national movement Setsuden ("saving electricity"). The movement caused a dramatic decrease in the country's energy consumption during the crisis and later inspired the Japanese government to pass a battery of policies focused on reducing the energy consumption of large companies and households.

**Table S1. Fukushima Disaster Narrative. Prior to the interaction phase in the network experiments, all participants read the four paragraph disaster explaining the causes of the Fukushima nuclear disaster and its consequences.**

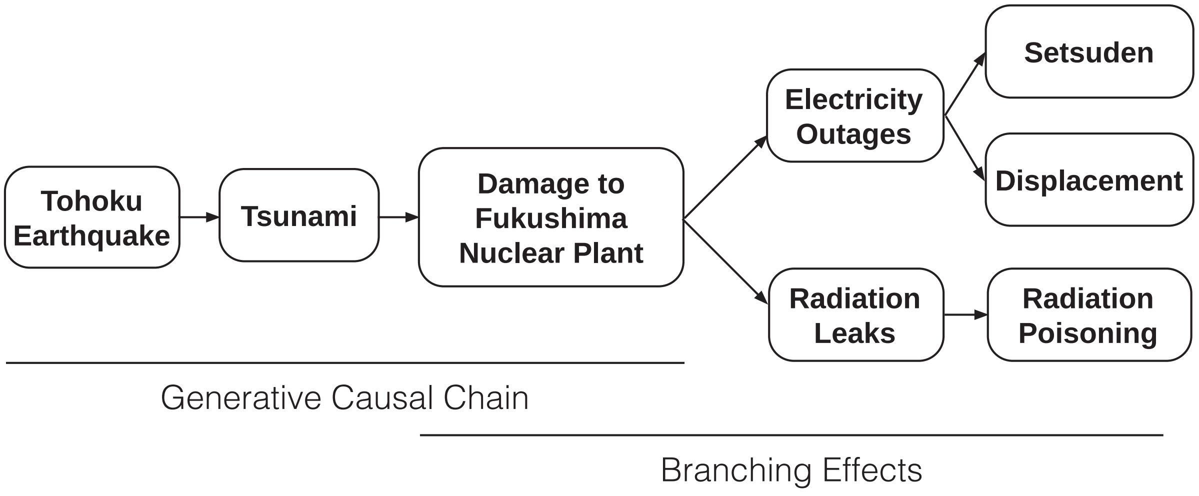


**Fig. S1. Causal model communicated by the Fukushima nuclear disaster *focal narrative*.** This diagram is just for illustration purposes, participants did not see this diagram. They read a four-paragraph narrative describing how the Tōhoku earthquake triggered a massive tidal wave that damaged the Fukushima Nuclear Power Plant, resulting in electricity outages, radiation leaks and poisoning, human displacement, and *Setsuden*, a national energy-saving holiday. The narrative text is available in Table S1 above.

## S6. Entropy dynamics of response distribution

Entropy encodes the overall coherence of a response distribution. High entropy values indicate more variation in responses, and low entropy indicates converging responses. As shown in Figure S2, we observe that the entropy approaches zero more quickly under the face-naming condition. To model these shifts, we fit a Gaussian-distributed GLM to predict the change in the entropy of the full response distribution across experimental runs. As shown in Supplemental Figure S2, a group's response entropy steadily decreased as a function of subsequent interactions in homogeneously-mixed face-naming networks ($\beta_{Trial} = -0.06$, 95% CI $[-0.07, -0.06]$), doing so more slowly in spatially-embedded face-naming networks ($\beta_{Trial:Spatial} = 0.04$, 95% CI $[0.03, 0.04]$). Entropy also decreased more slowly in groups matching hashtags ($\beta_{Trial:Hashtag} = 0.02$, 95% CI $[0.02, 0.03]$), suggesting that coherence emerges more slowly in situations involving media content with complex causal relations. Furthermore, the analysis revealed a significant three-way interaction between network structure, media content, and trial number, such that the rate of entropy change across network structures is greater in face-naming networks than hashtag-matching networks ($\beta_{Trial:Spatial:Hashtag} = -0.02$, 95% CI $[-0.03, -0.01]$).

## S7. Media content and network structure jointly shape response coordination between interacting participants

The group-level coherence findings described above replicate well-known findings in behavioral economics and sociology, suggesting that homogeneously-mixed networks better produce shared behaviors than spatially-embedded networks. However, the rate of convergence is mediated by the content of networked interaction. It is also worth considering that even when a spatially-embedded group does not adopt a shared behavior, participants could be aligning responses with their neighbors. As shown in Figures S3 and S4, participants form distinct semantic clusters resulting from separable social groups aligning on different behaviors (e.g., labeling a narrative with different causal content and topics). This "clustering effect" is evident in the clear horizontal bands (groups of rows) of semantically distinct responses forming in the spatially-embedded networks. While these figures only show a selected handful of runs, this phenomenon is common in spatially-embedded networks and is more pronounced in face-naming networks. This is because in the Name Game, complex background knowledge does not (1) slow the rate of coordinated responses (i.e., faster local response convergence,) and (2) cause separate social groups to align on a similar response without communications across groups (e.g., separate neighborhoods independently coordinating on a topic-label hashtag). This effect is a central reason for why network structure effects are more pronounced in cases where background knowledge is less pronounced in interaction.

We observed a steady increase in the number of participants who coordinated their responses in each of the experimental conditions, suggesting that subjects learn to match responses in both games and network structures. To test how the causal complexity of interaction affects the onset of local coordination, we fit a Bernoulli GLM to predict the probability a pair of nodes (i.e., partnered participants in a trial) coordinate responses by trial number with network structure and interaction content, while controlling for group size. At the beginning of an experiment, participants in the reference group (homogeneously-mixed face-naming networks of size 20) coordinate approximately 5% of the time ($\beta_{Intercept} = -2.83$, 95% CI $[-2.98, -2.70]$), with a coordination rate increasing by approximately 0.5% for each subsequent interaction ($\beta_{Trial} = 0.10$, 95% CI $[0.09, 0.10]$). Although participants in spatially-embedded networks coordinated more effectively than those in homogeneously-mixed networks ($\beta_{Spatial} = 1.46$, 95% CI $[1.29, 1.63]$), the adoption of shared behaviors in homogeneously-mixed networks is accompanied by faster learning to coordinate ($\beta_{Trial:Spatial} = -0.05$, 95% CI $[-0.05, -0.04]$). The media content affected coordination rates, as groups learned to coordinate face names more quickly than hashtags ($\beta_{Trial:Hashtag} = -0.04$, 95% CI $[-0.05, -0.04]$). The type of media contents also mediated the impact of network structure on coordination dynamics ($\beta_{Trial:Hashtag:Spatial} = 0.04$, 95% CI $[0.03, 0.05]$).

## S8. Statistical models for decision dynamics

To predict the number of participants following one of the four decision strategies in a given network structure and content interaction condition, we fit a Bayesian categorical regression model using `brms` (29) to predict decision type (Decision Type) based on trial number, content type (Face vs. Hashtag), structure (Homogeneous vs. Spatial), and group size. The model includes interactions between trial number and content type, and estimates log-odds for each non-reference decision type: Earlier Context (EC), Repeat Partner (RP), and Repeat Self (RS), relative to the baseline category Brand New (BN). Results show that hashtag content significantly reduces the likelihood of structured decision types (EC, RP, RS), while trial progression and spatial structure slightly increase the odds of RP and RS choices. Table S2 shows the full set of model parameters.

Trends discussed in the main text suggest that participants explore the hashtag space longer than face names, which makes it more difficult to coordinate responses and produce group consensus through narrative-language-belief coherence. To test this hypothesis we predicted the probability an individual coordinates with their partner given a each decision type. We fit a beta-distributed GLM to predict the probability that different strategies resulted in coordination as a function of decision type interacted with content while controlling for structure and group size. Coordination is coded 1 if two interacting partners provide the same response, otherwise 0.

As shown in Table S3, we found that participants are far less likely to coordinate a response if they repeat themselves in the hashtag condition than in the face name condition ($\beta_{Hashtag:RS} = -1.47$, 95% CI $[-1.77, -1.21]$). The effect of network structure on group-level outcomes is more pronounced in the face naming task because face names are more semantically interchangeable, thus more "repeatable", than hashtags, whose fitness is constrained by causal and semantic relations in the disaster narrative.

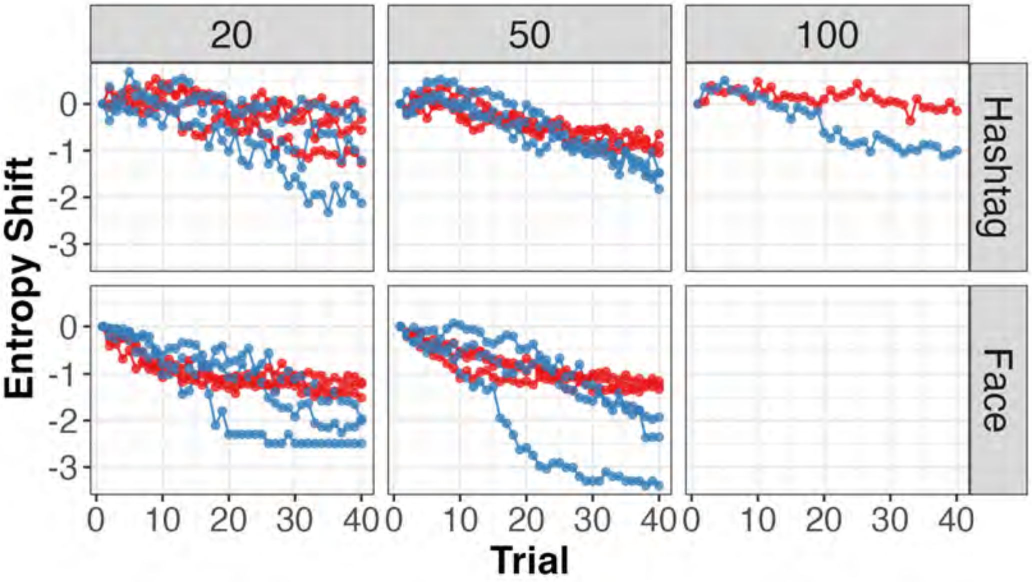


**Fig. S2.** Entropy shift of response distribution over course of interactions by group size (columns) and media content (rows). The entropy values represent the change in entropy in trial $t$ from the first trial, allowing normalization between group sizes (larger group sizes inherently have higher entropy due to larger distribution of responses).

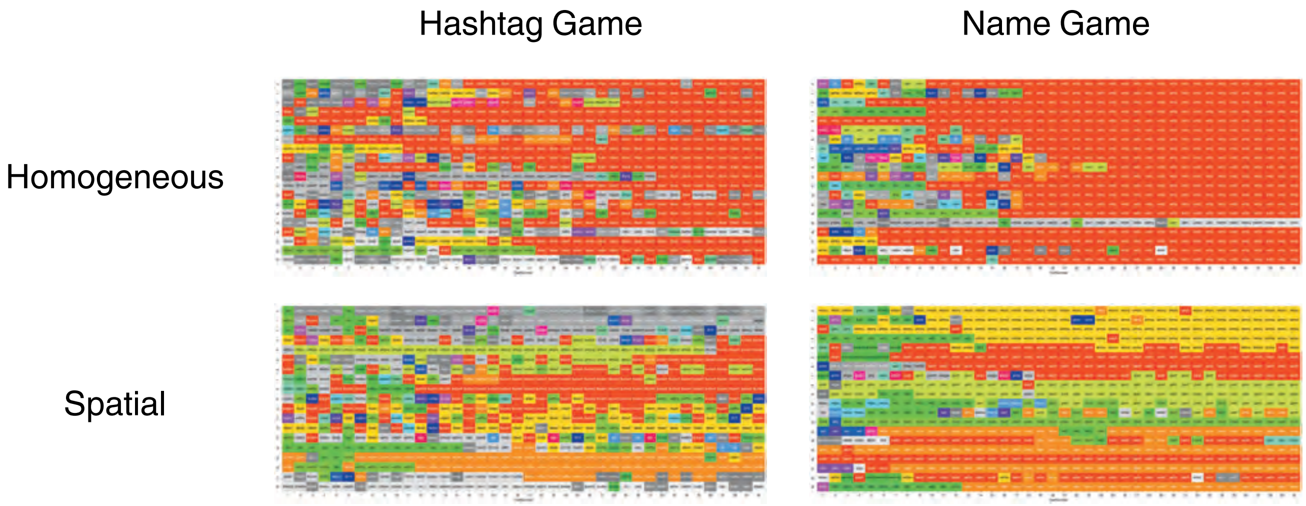


**Fig. S3. Colormaps of responses across a single $N = 20$ run of the Hashtag Game (Left) and Name Game (Right).** Top panel shows results for homogeneously-mixed networks. Spatially-embedded networks in the bottom panel. Color maps visualize group dynamics. Each cell represents an individual response of hashtags in a trial, rows represent individual participants, columns represent trials. Cells encode the first five letters of the generated hashtag, and colors are based on the hashtag label provided.

 

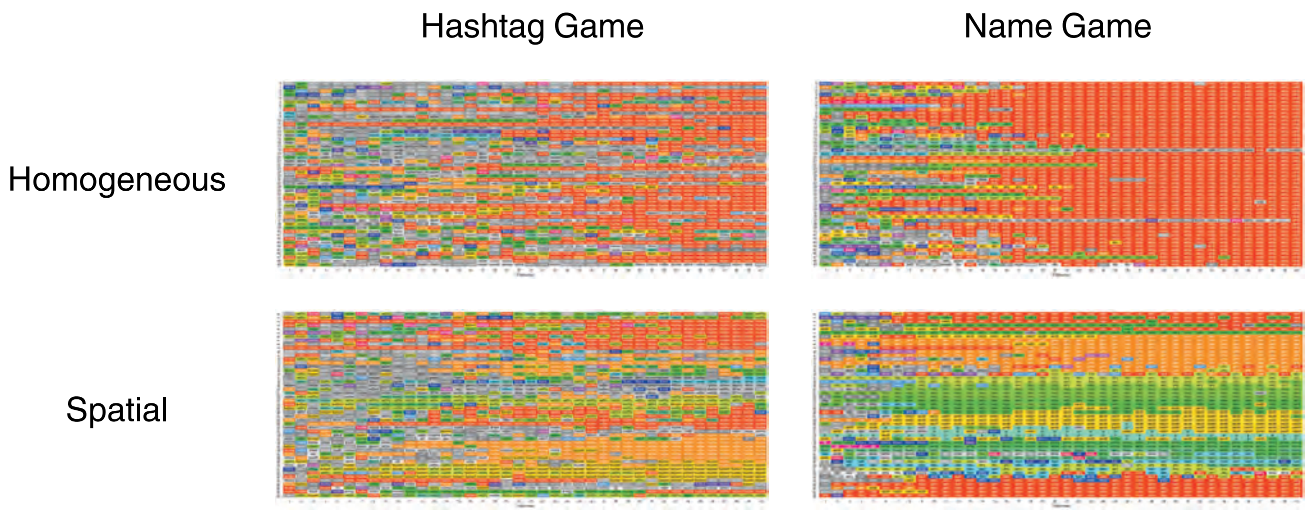


**Fig. S4. Colormaps of responses across a single $N = 50$ run of the Hashtag Game (Left) and Name Game (Right).** Top panel shows results for homogeneously-mixed networks. Spatially-embedded networks in the bottom panel. Color maps visualize group dynamics. Each cell represents an individual response of hashtags in a trial, rows represent individual participants, columns represent trials. Cells encode the first five letters of the generated hashtag, and colors are based on the hashtag label provided.

| Predictor | Estimate | SE | 95% CI Low | 95% CI High | Rhat |
|---|---|---|---|---|---|
| *Earlier Context (EC)* | | | | | |
| Intercept | -1.82 | 0.07 | -1.96 | -1.68 | 1.00 |
| Trial Number | 0.06 | 0.00 | 0.06 | 0.07 | 1.00 |
| Hashtag Content | -0.39 | 0.08 | -0.53 | -0.24 | 1.00 |
| Spatial Structure | 0.01 | 0.04 | -0.06 | 0.08 | 1.00 |
| Group Size | 0.01 | 0.00 | 0.01 | 0.01 | 1.00 |
| Trial × Hashtag | 0.01 | 0.00 | -0.00 | 0.01 | 1.00 |
| *Repeat Partner (RP)* | | | | | |
| Intercept | -1.17 | 0.06 | -1.30 | -1.05 | 1.00 |
| Trial Number | 0.04 | 0.00 | 0.03 | 0.05 | 1.00 |
| Hashtag Content | -1.04 | 0.07 | -1.18 | -0.90 | 1.00 |
| Spatial Structure | 0.24 | 0.04 | 0.17 | 0.31 | 1.00 |
| Group Size | 0.01 | 0.00 | 0.00 | 0.01 | 1.00 |
| Trial × Hashtag | 0.02 | 0.00 | 0.01 | 0.03 | 1.00 |
| *Repeat Self (RS)* | | | | | |
| Intercept | -0.34 | 0.05 | -0.44 | -0.24 | 1.00 |
| Trial Number | 0.09 | 0.00 | 0.09 | 0.10 | 1.00 |
| Hashtag Content | -1.37 | 0.06 | -1.49 | -1.26 | 1.00 |
| Spatial Structure | 0.29 | 0.03 | 0.24 | 0.35 | 1.00 |
| Group Size | 0.00 | 0.00 | -0.00 | 0.00 | 1.00 |
| Trial × Hashtag | -0.00 | 0.00 | -0.01 | 0.00 | 1.00 |

**Table S2. Population-level effects from categorical model predicting decision type (relative to baseline category).**

| Predictor | Estimate | SE | 95% CI Low | 95% CI High | Rhat |
|---|---|---|---|---|---|
| Intercept | -3.82 | 0.13 | -4.09 | -3.58 | 1.01 |
| Decision Type: EC | 2.05 | 0.15 | 1.77 | 2.35 | 1.01 |
| Decision Type: RP | 2.90 | 0.14 | 2.63 | 3.19 | 1.01 |
| Decision Type: RS | 3.72 | 0.13 | 3.47 | 4.00 | 1.01 |
| Content: Hashtag | 1.18 | 0.14 | 0.92 | 1.47 | 1.01 |
| Spatial Structure | 0.21 | 0.03 | 0.16 | 0.26 | 1.00 |
| Group Size | -0.00 | 0.00 | -0.00 | -0.00 | 1.00 |
| EC × Hashtag | -1.04 | 0.16 | -1.38 | -0.74 | 1.01 |
| RP × Hashtag | -1.31 | 0.15 | -1.63 | -1.03 | 1.01 |
| RS × Hashtag | -1.47 | 0.14 | -1.77 | -1.21 | 1.01 |

**Table S3. Posterior estimates from logistic regression predicting coordination as a function of decision type. Positive coefficients indicate increased odds of coordination.**

## S9. Initial distribution of responses illuminates participants' prior knowledge about media content

We examined participants' prior knowledge about media content using responses in the first trial of face naming and hashtag matching experimental runs. As shown in Figure S5, the prior for face names is more evenly distributed than that for hashtags, which shows that responses are more concentrated around broad topic labels (e.g., #NuclearDisaster) and causal/generative events (e.g., #Earthquake and #Tsunami) on the first trial in networked interaction. The prior distribution of possible responses influences how participants choose to explore background knowledge within a social context (i.e., responses sampled from prior interactions that yielded rewards) when coordinating with network neighbors. The high uncertainty in the distribution of face names promotes more reliance on learning from social context during networked interactions, which contributes to a quicker onset of group-level consensus.

## S10. Impact of media content on response coordination

Prominent game theory models used to study the emergence of coordinated behavior in networks typically account for the role utility representation plays in taking certain actions over others, but do not consider the impact of representational content connected to those utilities. Effective communication requires semantic and causal/categorical alignment across group members to facilitate social learning (e.g., converging on a shared language response or social norm). As shown in Figure S6, the top hashtags for coordination are a mix of semantic topics and cause-effect relational entities encoded by the narrative's situation model. Semantic and causal content constrain the response space and guide what constitutes an optimal response. Moreover, studies of naturally occurring hashtag behavior revealed that successful hashtags fall into one of two categories: focal hashtags, which serve to label posts with broad semantic topics to relate them to larger discussions and movements across an online network, and individualistic hashtags, which make the distribution of hashtags heavy-tailed, as they co-occur with focal hashtags while allowing users to signal personal narratives (13). In our experiment, the top three most successful hashtags align on the same discrete narrative entity, but have different string representations. Not only does causal complexity of the hashtag content make it more difficult to coordinate responses and raises the cognitive complexity of interaction, but also the role that

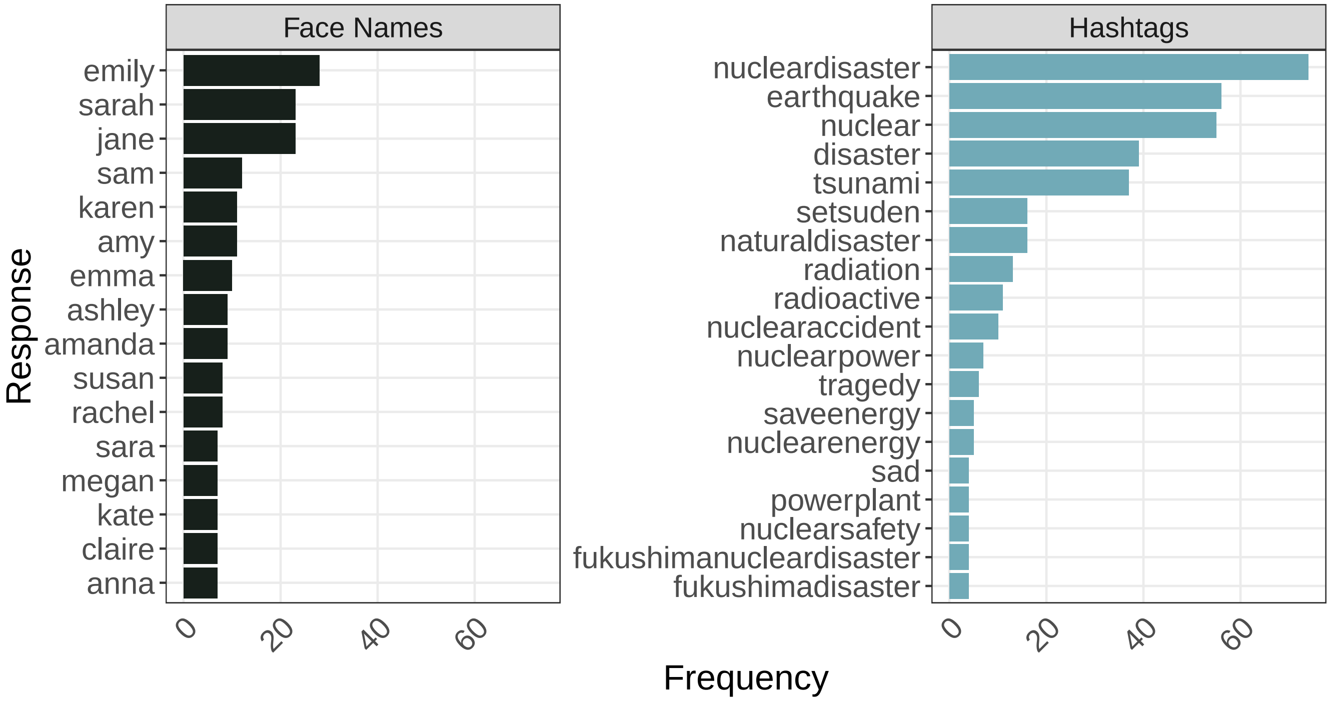


**Fig. S5.** Distribution of the most common responses in the first trial of face naming and hashtag matching experimental runs. In the first trial, face name responses exhibit a widely dispersed distribution, while hashtag responses show a more skewed distribution with increased frequency of hashtags that describe broad topics (e.g., Nuclear Disaster) and causal topics expressed in the disaster narrative.

additional appending of semantic content to signal a personal view (representation of narratives) has on hashtag coordination.

## S11. Modeling causal language identified in personal narratives

Priniski, Verma, & Morstatter (2023) (30) developed a causal language identification model that extracts causal claims expressed in text documents. The model identifies spans of words that serve as input to explicitly stated causal relations. Both the cause and effect events, and the underlying causal relation (i.e., a causal trigger) are explicitly stated for the algorithm as prior knowledge to identify the causal claim. The extracted claims are then co-referenced based on embeddings of the identified entities as computed by a fine-tuned RoBERTa-XL transformer model (31, 32), to produce clusters of semantically similar topics, termed "causal topics." The model additionally encodes the direction of the stated causal relationships linking any two topics. To extract causal relationships expressed in the personal narratives, we used the causal language model to analyze all personal narratives generated by participants before and after networked interaction. The model identified documents expressing explicit causal claims (i.e., a cause-effect relationship) and clustered the claims based on their semantic content. See Table S4 for ten representative tweet-like personal narratives.

As shown in Table S5, the unsupervised model identified 20 causal topics, with topics relating to the events described in the narrative (e.g., Earthquake, Tsunami, Nuclear Disaster), in addition to broad semantic-level topics not explicitly expressed in the narrative (i.e., a general topic for Natural Disaster which is a superordinate category of earthquake/tsunami in the narrative). Each document with an identified causal relation received a cause cluster label and an effect cluster label, with some clusters relating more to causes and some more to effects. As shown in Supplemental Table S5, to reduce noise in the statistical analysis of causal language shift, we remapped the unsupervised topic labels onto the Fukushima narrative's causal model (see Figure S1), collapsing redundant topics as necessary. Specifically, the causal language model identified twenty distinct topics, which mapped onto each of the nine discrete narrative entities (9 causal events plus one topic sentence).

**A. Causal language change in personal narratives following network interaction.** As shown in Figure S7, and discussed in the main part of the manuscript, the distributions of the shifts in the amount of causal language for each participant (the number of causal claims generated after interaction subtracted by the number of causal claims generated before interaction) are zero-inflated. A zero-inflated model assumes that a separate data generating process produces a distribution of zero values alongside the set of positive values, thus a zero-inflated model learns separate sets of statistical parameters: one set for the process that produces a zero value; one set for the process that produces a non-zero value.

We fit a hurdle Gaussian model to predict the shift in the number of causal claims that a participant produced after network interaction (difference = number of causal claims produced after interaction - number of causal claims produced before interaction). The hurdle Gaussian model consists of a logistic classification step to identify personal narratives without causal claims, and then a Gaussian distribution estimating the difference scores for the remaining documents (33). We examined how network structure and content in networked interaction impact the difference scores.

The hurdle Gaussian model reveals that around 49% of the participants did not show a change in the number of causal claims in personal narratives after networked interaction ($hu = .49$, 95% CI [0.46, 0.52]). It is worth noting that of the 49% of participants who did not show a shift in the amount of causal relations expressed, not all of these participants did not update the causal content of their personal narratives following networked interaction. 33% of these participants explicitly mentioned causal relationships before and after networked interactions, but the number of relationships was consistent across interaction phases. These individuals were identified by zeros in the hurdle model. In the claim level analysis in the main text we analyze how causal language shifted for these participants. It is important to evaluate both analyses as composing a broader whole.

The intercept of the Gaussian linear model component equals the mean change in the number of causal claims for participants placed in homogeneously-mixed face-naming networks, which is not credibly different from zero ($\beta_0 = .21$, 95% CI [-0.12, 0.53]). In line with our prediction that media content will have a main effect on the generation of causal content in personal narratives, we found a significant effect of hashtag interaction on change in causal language ($\beta_{Hashtag} = .45$, 95% CI [0.01, 0.87]).

**B. Significantly shifted causal relations following network interaction.** As discussed in the main text, we performed a t-test to identify significantly shifted causal relationships expressed in personal narratives following network interactions. We applied a False Discover Rate by only considering relations in the causal model.

Tables S6, S7, S8, S9 show the list of shifted causal relations using the FDR correction (i.e., causal relations that map onto causal model, plus relations with broader topics including Natural Disaster).

## S12. Agent response strategy updating and interaction patterns in CAA model

In the main text we introduce an agent-based model of context aware agents (CAA). Agents in the model update their sampling strategies adaptively from both background prior knowledge and interaction history distributions, by following an updating procedure that weighs rewarded trials against non-rewarded trials when selecting a given decision strategy. Given the reward incentive for coordinating with networked neighborhoods, we can assess how network structure and content priors jointly impact the onset of group coherence.

On a given trial, simulated agents generate a hashtag by combining background knowledge about interaction media and social context through a *learning parameter*, $\alpha$, which determines how slow individuals move from sampling prior distributions of responses to sampling responses observed in social context. The *self-preference* parameter, $\gamma$, determines an individual's

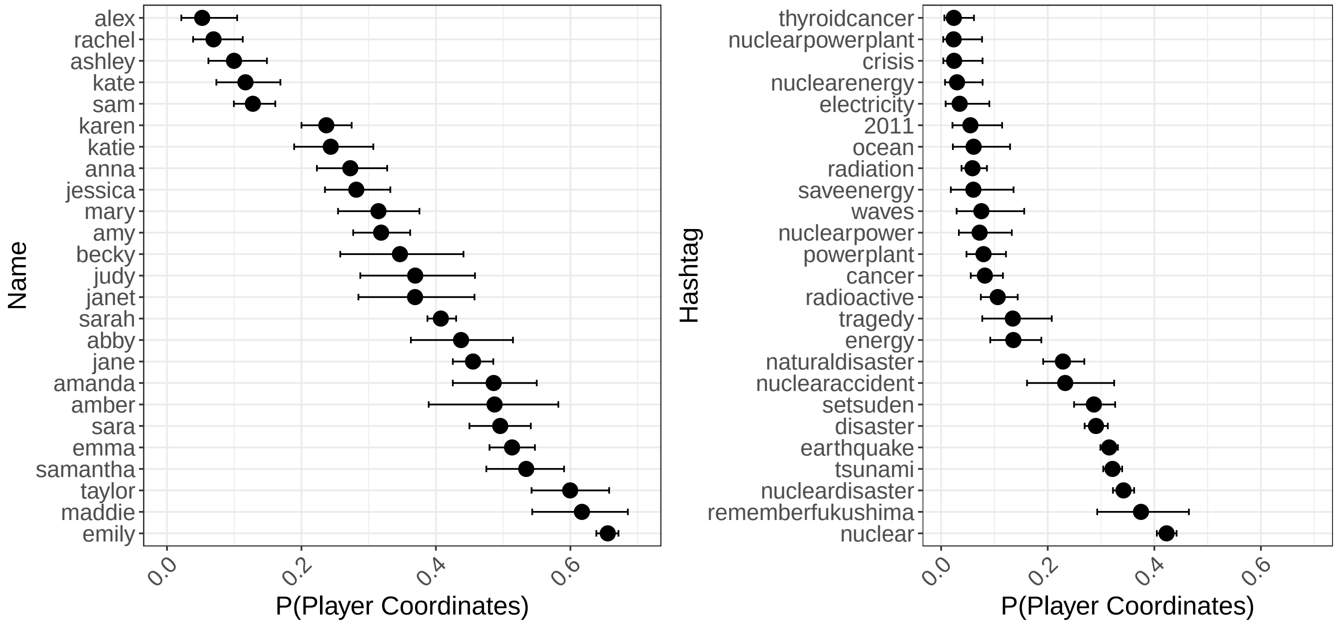


**Fig. S6.** Probability distribution of player coordination **Left:** Probability of a participant coordinating given a sampled face. The analysis focuses on trials that contained one of the top 25 faces.**Right:** Probability of a participant coordinating given a sampled hashtag. The analysis focuses on trials that contained one of the top 25 hashtags.

| ID | Phase | Response | Content | Structure |
|---|---|---|---|---|
| 98496 | Preinteraction | japan went through one of the most gruesome natural disasters of all time and we can see its population still facing its consequences. what came out of it, their national movement setsuden; however, is a lesson everyone can learn and implement | Hashtag | Spatial |
| 98496 | Postinteraction | the catastrophe in japan was an act of nature that the the population of japan is still recovering from. however, the national movement - setsuden- is something we can all learn from | Hashtag | Spatial |
| 89947 | Preinteraction | fukushima nuclear disaster will be with us for many years to come. fukushima nucleardisaster earthquake japan tsunami 2011 nuclearreactor pacificocean california coast radioactive | Hashtag | Spatial |
| 89947 | Postinteraction | nuclear disasters last longer and cause lifelong consequences. | Hashtag | Spatial |
| 85834 | Preinteraction | fukushima nuclear event caused a displacement of over 150k people but the affects can be felt worldwide all the way to united states. causing thyroid cancer in some japanese girls. reducing energy consumption has been the focus in japanese new policies. | Hashtag | Spatial |
| 85834 | Postinteraction | japan just gone through a earthquake tsunami and nuclear plant crisis of fukushima. our hearts are with them. | Hashtag | Spatial |
| 57561 | Preinteraction | japan needs our help! we must do what we can to offer resources to those affected. this news breaks my heart and i hope that everyone is okay. sending thoughts and prayers to those affected | Hashtag | Spatial |
| 57561 | Postinteraction | we need to help save japan because they are in trouble | Hashtag | Spatial |
| 54392 | Preinteraction | this day in history-march 11, 2011 the most powerful earthquake ever recorded hit japan. it triggered a tsunami that had waves up to 130 ft tall! it caused damage to a nuclear plant. | Hashtag | Homogeneous |
| 54392 | Postinteraction | biggest earthquake in japans history occurred in march 2011. it caused tsunami waves as tall as 130ft. it hit a nuclear plant and released radioactive waves. it caused thyroid cancer in baby girls. | Hashtag | Homogeneous |
| 10566 | Preinteraction | there is a lot of pain and sorrow from what happened at the plant in fukushima. a tsunami wiped out the city and caused damage to a nuclear power plant. it displaced 156000 people. then there was a movement to descrease electric use in japan | Hashtag | Homogeneous |
| 10566 | Postinteraction | there was a devistating tsunami wave that hit japan which was caused by a earthquake. it displaced 156,000 people. the effects are still being felt of this even til this day | Hashtag | Homogeneous |
| 12045 | Preinteraction | i hope the world never forgets the disaster at fukushima. after a massive earthquake triggered an even more massive tsunami, the fukushima nuclear plant was irreperably damaged and leaked radioactive particles for hundreds of miles. we're still quantifying its impact today. | Hashtag | Homogeneous |
| 12045 | Postinteraction | in 2011, japan's relationship with nuclear energy changed forever when the fukushima daiichi power plant experienced catastrophic damage in the aftermath of a historic earthquake and subsequent tsunami. nuclear waste leaked and caused permanent damage | Hashtag | Homogeneous |
| 16728 | Preinteraction | 15 years ago the fukishima plant disaster occurred. remembering all of the victims today, but also remembering the japanese effort to save energy that led to sweeping reforms! | Hashtag | Homogeneous |
| 16728 | Postinteraction | the fukishima meltdown was a tragic happening that occurred when a earthquake driven tsunami hit a nuclear power plant. the effects are still felt to this day. a silver lining was japanese energy reform. | Hashtag | Homogeneous |

**Table S4. Example pre- and post-interaction personal narratives. Narratives are from participants sampled across Hashtag Games. These examples show cases of when participants shift causal language in personal narratives after. For example, by mentioning the narrative's causal chain explaining the cause of the nuclear disaster.**

| Remapped | Narrative Entity | Unsupervised | ID | Keywords |
|---|---|---|---|---|
| Earthquake | Yes | (Tohoku) Earthquake | 15 | tohoku earthquake, the 2011 tohoku earthquake |
| | | Earthquake | 1 | massive earthquake, the earthquake |
| Tsunami | Yes | Tsunami | 6 | tsunami, a tsunami, the tsunami |
| | | Tsunami (misspelt) | 18 | a large tsunami, a tsunami |
| | | Waves | 11 | a tidal wave, 130 foot waves, 130 foot tsunami |
| Nuclear Disaster | Yes | Nuclear Disaster | 0 | nuclear disaster, fukushima nuclear disaster |
| | | Damage | 7 | the damage, intense damage, widespread damage |
| Electricity Outage | Yes | Change (Loss) | 9 | reducing, loss, outage |
| Radiation Leaks | Yes | Radiation | 3 | radiation, radioactive isotopes, particles |
| Setsuden | Yes | Energy Movement | 2 | Setsuden, energy crisis, conserving electricity |
| Poisoning | Yes | Health Issues | 10 | health concerns, health problems, many illnesses |
| | | Cancer | 19 | thyroid cancer, cancer, thyroid issues |
| Displacement | Yes | Displacement | 14 | displaced, the displacement, displaced people |
| Disaster | No | Disaster | 4 | a disaster, the disaster, many disasters |
| Effects | No | Effects | 5 | harm, environmental damage, devastating effects |
| Issues | No | Issues | 8 | problems, issues, fails, many problems |
| Terrible Event | No | Terrible Event | 12 | the event, terrible events, cataclysmic events |
| Destruction | No | Destruction | 13 | destruction, destroyed, the devastation |
| Incident | No | Incident | 16 | an accident, devastating accident, an incident |
| Natural Disaster | No | Natural Disaster | 17 | a natural disaster, this natural disaster |
| No Cluster | No | No Cluster | -1 | Not Applicable |

**Table S5. Causal topics identified by the causal language model. The causal topic model identified 20 topics plus a catch-all “no cluster" topic. Each of the topics aligned with at least one of the narrative entities described in the Fukushima disaster materials, along with additional semantic topics. In some cases, the model identified separate clusters that aligned with the same narrative entity described in the materials (unsupervised topics), which we mapped to the underlying narrative (remapped). Statistical analyses were performed on the remapped topics. The keywords represent the most common entities in each of the unsupervised topics.**

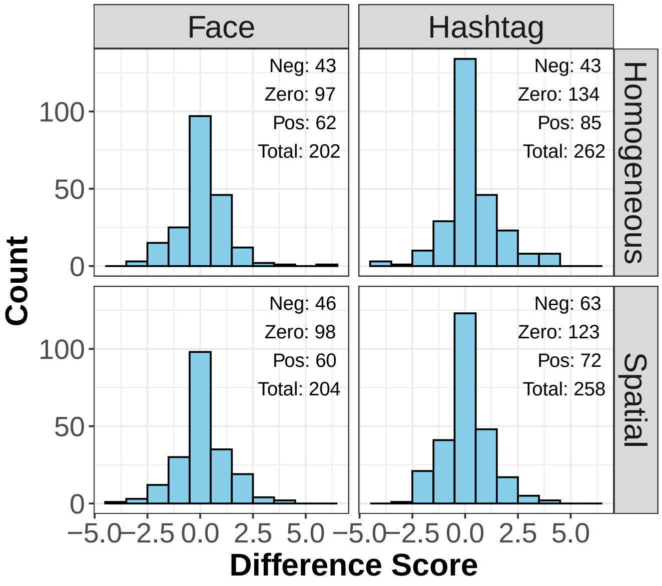


**Fig. S7. Distribution of changes in the amount of causal language produced in each experimental condition.** The text in the top right of each panel describes the number of documents exhibiting a negative shift (more causal claims before network interaction compared to after), zero shift (the same amount of identified causal relations before and after), and positive shift (more causal relations after network interaction than before). For each condition, the shift is zero-inflated, indicating that many participants did not exhibit a shift in causal language.

| Structure | Content | Cause Topic | Effect Topic | Estimate | P value | Conf. Low | Conf. High | FDR |
|---|---|---|---|---|---|---|---|---|
| Homogeneous | Hashtag | Tsunami | Nuclear Disaster | 0.0958 | 0.0015 | 0.0372 | 0.1545 | 0.0106 |
| Homogeneous | Hashtag | Earthquake | Tsunami | 0.0898 | 0.0050 | 0.0275 | 0.1522 | 0.0176 |
| Homogeneous | Hashtag | Natural Disaster | Nuclear Disaster | 0.0240 | 0.0452 | 0.0005 | 0.0474 | 0.0790 |
| Homogeneous | Hashtag | Tsunami | Destruction | 0.0240 | 0.0452 | 0.0005 | 0.0474 | 0.0790 |
| Homogeneous | Hashtag | Radiation | Health Issues | -0.0240 | 0.2069 | -0.0613 | 0.0134 | 0.2896 |
| Homogeneous | Hashtag | Nuclear Disaster | Change (Loss) | -0.0120 | 0.3188 | -0.0356 | 0.0117 | 0.3719 |
| Homogeneous | Hashtag | Nuclear Disaster | Radiation | 0.0060 | 0.7824 | -0.0368 | 0.0487 | 0.7824 |

**Table S6. Cause and effect relations (Cause topic $\rightarrow$ Effect Topic) increased in personal narratives written after homogeneously-mixed hashtag matching**

| Structure | Content | cause_topic | effect_topic | estimate | p.value | conf.low | conf.high | fdr |
|---|---|---|---|---|---|---|---|---|
| Spatial | Hashtag | Earthquake | Tsunami | 0.0674 | 0.0139 | 0.0139 | 0.1210 | 0.0486 |
| Spatial | Hashtag | Tsunami | Nuclear Disaster | 0.0730 | 0.0089 | 0.0185 | 0.1276 | 0.0486 |
| Spatial | Hashtag | Nuclear Disaster | Change (Loss) | -0.0112 | 0.3187 | -0.0334 | 0.0109 | 0.5577 |
| Spatial | Hashtag | Radiation | Health Issues | 0.0169 | 0.2580 | -0.0125 | 0.0462 | 0.5577 |
| Spatial | Hashtag | Tsunami | Destruction | 0.0056 | 0.5652 | -0.0136 | 0.0249 | 0.7912 |
| Spatial | Hashtag | Nuclear Disaster | Radiation | 0.0056 | 0.7400 | -0.0277 | 0.0390 | 0.8632 |
| Spatial | Hashtag | Natural Disaster | Nuclear Disaster | 0.0000 | 1.0000 | -0.0222 | 0.0222 | 1.0000 |

**Table S7. Cause and effect relations (Cause topic → Effect Topic) increased in personal narratives written after spatially-embedded hashtag matching**

| Structure | Content | cause_topic | effect_topic | estimate | p.value | conf.low | conf.high | fdr |
|---|---|---|---|---|---|---|---|---|
| Homogeneous | Face | Radiation | Health Issues | 0.0511 | 0.0077 | 0.0138 | 0.0884 | 0.0538 |
| Homogeneous | Face | Nuclear Disaster | Radiation | -0.0292 | 0.1027 | -0.0644 | 0.0059 | 0.3594 |
| Homogeneous | Face | Tsunami | Nuclear Disaster | -0.0365 | 0.2266 | -0.0959 | 0.0229 | 0.5286 |
| Homogeneous | Face | Nuclear Disaster | Change (Loss) | 0.0146 | 0.3191 | -0.0143 | 0.0435 | 0.5584 |
| Homogeneous | Face | Change (Loss) | Energy Movement | -0.0073 | 0.6564 | -0.0397 | 0.0251 | 0.6564 |
| Homogeneous | Face | Earthquake | Tsunami | 0.0146 | 0.5948 | -0.0396 | 0.0688 | 0.6564 |
| Homogeneous | Face | Tsunami | Destruction | -0.0073 | 0.5656 | -0.0324 | 0.0178 | 0.6564 |

**Table S8. Cause and effect relations (Cause topic $\rightarrow$ Effect Topic) increased in personal narratives written after homogeneously-mixed face-name matching**

| Structure | Content | cause_topic | effect_topic | estimate | p.value | conf.low | conf.high | fdr |
|---|---|---|---|---|---|---|---|---|
| Spatial | Face | Earthquake | Tsunami | 0.0863 | 0.0068 | 0.0242 | 0.1485 | 0.0479 |
| Spatial | Face | Nuclear Disaster | Radiation | -0.0144 | 0.3191 | -0.0428 | 0.0141 | 0.8111 |
| Spatial | Face | Tsunami | Nuclear Disaster | 0.0288 | 0.3476 | -0.0320 | 0.0892 | 0.8111 |
| Spatial | Face | Natural Disaster | Nuclear Disaster | -0.0072 | 0.6564 | -0.0391 | 0.0247 | 0.8636 |
| Spatial | Face | Radiation | Health Issues | 0.0072 | 0.7402 | -0.0356 | 0.0500 | 0.8636 |
| Spatial | Face | Tsunami | Destruction | 0.0072 | 0.5656 | -0.0175 | 0.0319 | 0.8636 |
| Spatial | Face | Nuclear Disaster | Change (Loss) | 0.0000 | 1.0000 | -0.0349 | 0.0349 | 1.0000 |

**Table S9. Cause and effect relations (Cause topic $\rightarrow$ Effect Topic) increased in personal narratives written after spatially-embedded face-name matching**

intrinsic preference for repeating themselves over their partner. Specifically, these parameters weigh which decision strategies an agent adopts to sample a response, which impacts what information is brought into social context (see Figure 4A).

As shown in Figure (SM), context priors are fit to experimental data (i.e., responses across all experimental first trials), and where an individual agent's probabilities of sampling a specific strategy on a given trial is calculated as follows:

$$P(\text{BN}) = \frac{\alpha}{\alpha + T + C}, \quad P(\text{EC}) = (1 - P(\text{BN})) \cdot \frac{p_{ec} + 3}{p_{ec} + p_{rs} + p_{rp} + 20},$$
$$P(\text{RP}) = (1 - P(\text{BN}) - P(\text{EC})) \cdot \gamma \cdot \frac{p_{rp} + 1}{p_{rp} + p_{rs} + 2}, \quad P(\text{RS}) = 1 - P(\text{BN}) - P(\text{EC}) - P(\text{RP}),$$

where $T$ is the current time-step, $\alpha \in (0, \infty)$ is termed as "learning parameter" which dictates the speed of the network to move away from using the "brand new" strategy BN, $C$ is the number of points scored per individual weighted by the time those points were scored, $p_{ec}, p_{rs}$, and $p_{rp}$ are the time-weighted points using earlier context, repeat self, and repeat partner decision strategies, respectively, and $\gamma \in [0, 1]$ is the self-valuation parameter, conferring a possible proclivity to favor repeating yourself over repeating your partner. A diagram illustrating the structure of this decision pipeline is shown in S8.

To account for the experimental data using our CAA model, we assume that all agents pull a learning and self preference parameter from a normal distribution centered at the fitted $\alpha$ value (see below) with standard deviation equal to $\alpha/2$. We uncovered the optimized learning parameter by minimizing the $\ell^2$-distance on the entropy time series between the simulated and experimental results.

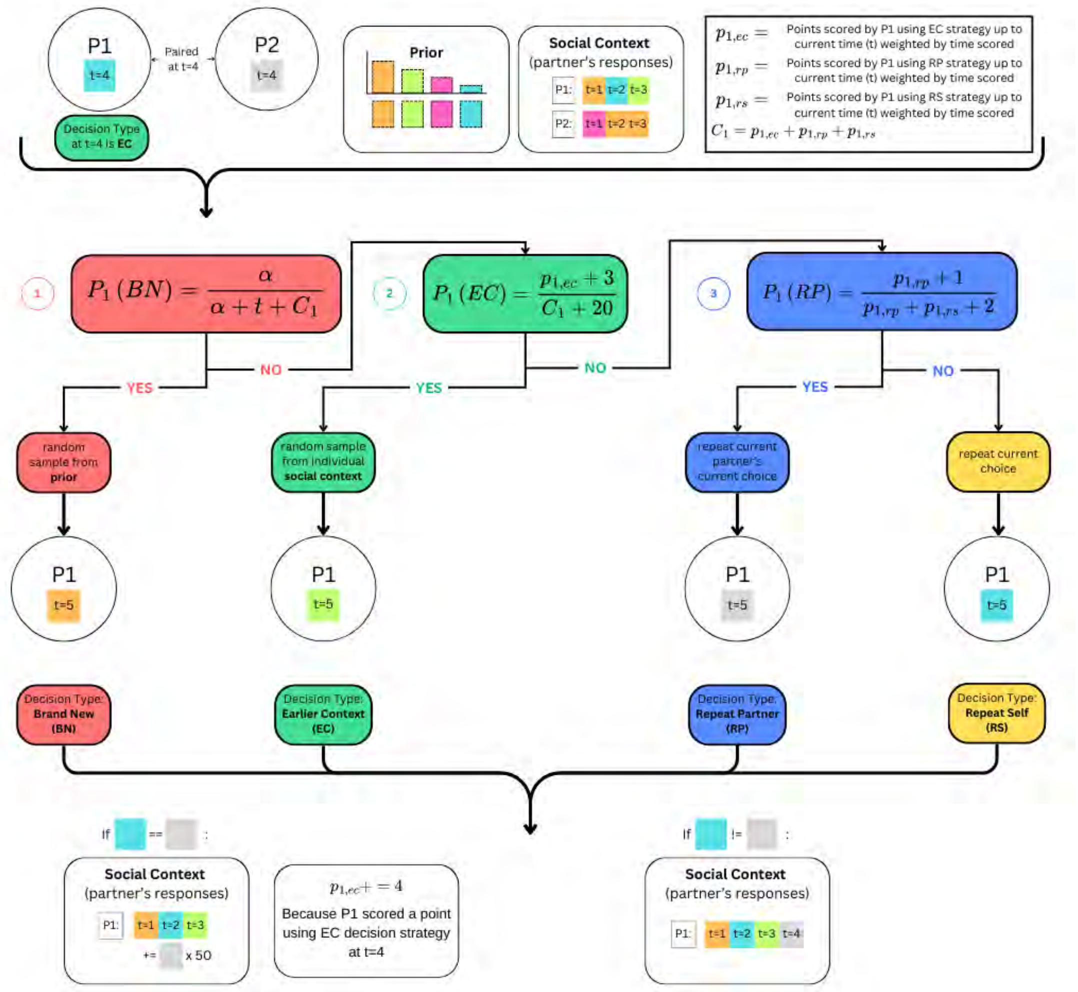


**Fig. S8. Illustration of Decision Making Model**. This diagram illustrates the decision-making flow for P1 at time t=5 given the input information. Agent decisions are independent. While probabilities are necessarily dependent on the *responses* of other agents in the network, they are not at all dependent on the *decision strategies* of the other individuals. This is a necessary component of the model to facilitate appropriate parameter optimization.

## S13. Pseudocode for computational models

The pseudocode for the two computational decision-making models tested in this paper is shown below. The code for both models is on the project's repository on the GitHub at github.com/jpriniski/NetCom.

**Algorithm 1** Centola Decision-Making Algorithm

**for** each round $t$ in number of rounds **do**
  **for** each pair (agent1, agent2) in pairings **do**
    Randomly assign speaker and hearer roles to agent1 and agent2
    **if** speaker's current response is in hearer's vocabulary **then**
      speaker's vocabulary $\leftarrow$ {speaker's response}
      hearer's vocabulary $\leftarrow$ {speaker's response}
    **else**
      Add speaker's response to hearer's vocabulary
      agent1's new response $\leftarrow$ random selection from agent1's vocabulary
      agent2's new response $\leftarrow$ random selection from agent2's vocabulary

**Algorithm 2** CAA Decision-Making Algorithm

**for** each round $t$ in number of rounds **do**
  **for** each pair (agent1, agent2) in pairings **do**
    **if** agent1's response = agent2's response **then**
      Update each agent's scores (and relevant decision-type scores)
      **for** each agent **do**
        decision type is BN with probability $\frac{\alpha}{\alpha+t+\text{time-weighted context points}}$ ▷ time-weighted context points = $\sum_t$ context sampling resulted in point
        **if** decision type is BN **then**
          agent's new response $\leftarrow$ random sample from experiment prior
        **else**
          decision type is EC with probability $\frac{\text{EC points} + 3}{\text{time-weighted context points} + 20}$
          **if** decision type is EC **then**
            agent's new response $\leftarrow$ random sample from agent's context
          **else**
            decision type is RS with probability $\frac{\text{RS points} + 1}{\text{RS points} + \text{RP points} + 2}$
            **if** decision type is RS **then**
              agent's new choice $\leftarrow$ agent's current choice
            **else** ▷ decision type is RP
              agent's new choice $\leftarrow$ partner's current choice
              **if** agent1's response = agent2's response **then**
                **for** each agent **do**
                  Add $50 \times t$ instances of partner's response to their context ▷ agents favor responses that have scored them points

                  **for** each agent **do**
                    Add $t$ instances of partner's response to their context

## S14. Optimizing model hyper-parameters with human data

Participants adopted different response strategies in different interaction content conditions which impacted the onset of group-level consensus (i.e., participants were more likely to leverage previously encountered and generated responses in the face naming condition than in the hashtag matching condition). Our computational models included an $\alpha$ parameter that constrained how often an agent samples new responses versus re-samples previous responses over the course of network interactions. As described in S12, each agent used a unique learning parameter value sampled from a normal distribution centered at the estimated $\alpha$ value with standard deviation equal to $\alpha/2$.

As shown in Figure S9, we plot the average distance between simulated and experimental entropy vectors for each of the four experiment types while controlling for network size. We see that the best-fitting exploration parameter for face-naming networks ($\alpha = 0.40$), is much lower than the best-fitting exploration parameter for hashtag-matching networks ($\alpha = 4.90$). Furthermore, for these alpha parameters, there is no dramatic difference across network structures (up versus down pointing arrows). These results suggest that interaction content is the salient factor driving exploration strategies in human groups, and lends support to the cognitive complexity hypothesis that individuals are slower to exploit environmental regularities in complex interaction tasks which limits the onset of group-level consensus. The difference in $\alpha$ values for these conditions reveals how exploring background knowledge longer (higher alpha) mediates the effect of network structure on group dynamics, as evident by the increased distance between red and blue lines at trial 40 in the right two panels (CAA model) of Figure ??B.

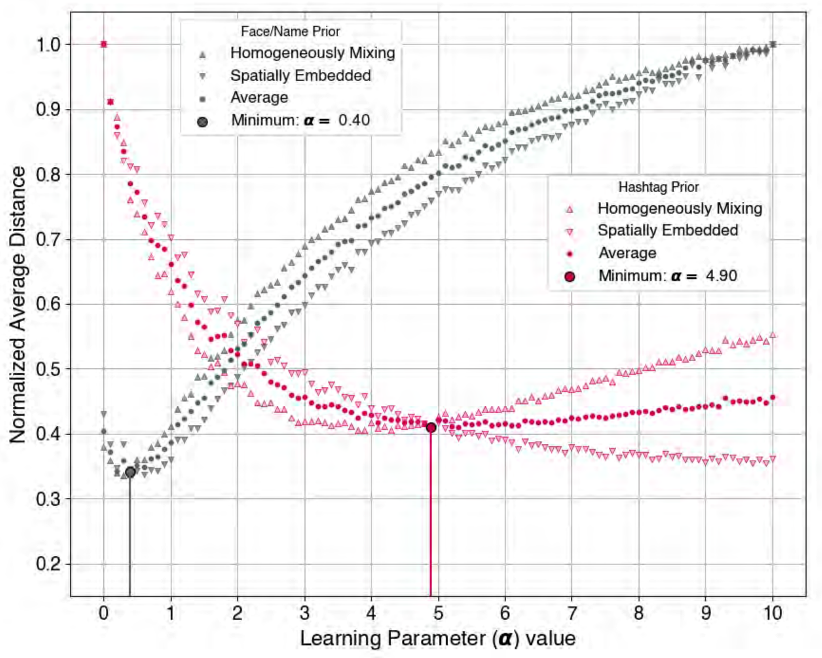


**Fig. S9.** Normalized average distance in the group level response entropy between the CAA model and experimental runs conditioned on a specified value of $\alpha$, which constrains how much agents re-sample previously encountered and generated responses on a present trial. Model fits for face-naming groups is illustrated in gray, while model fits for hashtag-matching groups is in pink.

## References

1. RX Hawkins, ND Goodman, RL Goldstone, The emergence of social norms and conventions. *Trends Cogn. Sci.* **23**, 158–169 (2019).
2. Y Du, JZ Leibo, U Islam, R Willis, P Sunehag, A review of cooperation in multi-agent learning. *arXiv preprint arXiv:2312.05162* (2023).
3. D Centola, A Baronchelli, The spontaneous emergence of conventions: An experimental study of cultural evolution. *Proc. Natl. Acad. Sci.* **112**, 1989–1994 (2015).
4. L Wittgenstein, *Philosophical Investigations.* (John Wiley & Sons, West Sussex, UK), (2009).
5. D Lewis, *Convention: A Philosophical Study.* (John Wiley & Sons), (2008).
6. D Guilbeault, A Baronchelli, D Centola, Experimental evidence for scale-induced category convergence across populations. *Nat. Commun.* **12**, 327 (2021).

7. D Barkoczi, M Galesic, Social learning strategies modify the effect of network structure on group performance. *Nat. Commun.* **7**, 13109 (2016).
8. A Banerjee, E Breza, AG Chandrasekhar, M Mobius, Naive learning with uninformed agents. *Am. Econ. Rev.* **111**, 3540–3574 (2021).
9. AG Chandrasekhar, H Larreguy, JP Xandri, Testing models of social learning on networks: Evidence from two experiments. *Econometrica* **88**, 1–32 (2020).
10. D Centola, The spread of behavior in an online social network experiment. *Science* **329**, 1194–1197 (2010).
11. D Centola, The network science of collective intelligence. *Trends Cogn. Sci.* **26**, 923–941 (2022).
12. MJ Gelfand, S Gavrilets, N Nunn, Norm dynamics: Interdisciplinary perspectives on social norm emergence, persistence, and change. *Annu. Rev. Psychol.* **75**, 341–378 (2024).
13. K Booten, Hashtag drift: Tracing the evolving uses of political hashtags over time in *Proceedings of the 2016 CHI Conference on Human Factors in Computing Systems.* (ACM), pp. 2401–2405 (2016).
14. Z Papacharissi, Affective publics and structures of storytelling: Sentiment, events and mediality. *Information, Commun. & Soc.* **19**, 307–324 (2016).
15. RA Zwaan, MC Langston, AC Graesser, The construction of situation models in narrative comprehension: An event-indexing model. *Psychol. Sci.* **6**, 292–297 (1995).
16. RA Zwaan, JP Magliano, AC Graesser, Dimensions of situation model construction in narrative comprehension. *J. Exp. Psychol. Learn. Mem. Cogn.* **21**, 386 (1995).
17. RA Zwaan, GA Radvansky, Situation models in language comprehension and memory. *Psychol. Bull.* **123**, 162 (1998).
18. RA Zwaan, Conspiracy thinking as situation model construction. *Curr. Opin. Psychol.* **47**, 101413 (2022).
19. K Giaxoglou, #JeSuisCharlie? Hashtags as narrative resources in contexts of ecstatic sharing. *Discourse, Context. & Media* **22**, 13–20 (2018).
20. P Dawson, Hashtag narrative: Emergent storytelling and affective publics in the digital age. *Int. J. Cult. Stud.* **23**, 968–983 (2020).
21. G Yang, Narrative agency in hashtag activism: The case of #BlackLivesMatter. *Media Commun.* **4**, 13–17 (2016).
22. PN Howard, MM Hussain, *Democracy's Fourth Wave?: Digital Media and the Arab Spring.* (Oxford University Press, Oxford), (2013).
23. M Zappavigna, Searchable talk: The linguistic functions of hashtags. *Soc. Semiot.* **25**, 274–291 (2015).
24. YR Lin, D Margolin, B Keegan, A Baronchelli, D Lazer, #Bigbirds never die: Understanding social dynamics of emergent hashtags in *Proceedings of the International AAAI Conference on Web and Social Media.* Vol. 7, pp. 370–379 (2013).
25. E Cunha, et al., Analyzing the dynamic evolution of hashtags on Twitter: a language-based approach in *Proceedings of the workshop on language in social media (LSM 2011).* pp. 58–65 (2011).
26. ML Avolio, et al., A comprehensive approach to analyzing community dynamics using rank abundance curves. *Ecosphere* **10**, e02881 (2019).
27. LM Hallett, et al., CODYN: An R package of community dynamics metrics. *Methods Ecol. Evol.* **7** (2016).
28. R McElreath, *Statistical Rethinking: A Bayesian Course with Examples in R and Stan.* (Chapman and Hall/CRC, New York), (2016).
29. PC Bürkner, brms: An R package for Bayesian multilevel models using Stan. *J. Stat. Softw.* **80**, 1–28 (2017).
30. JH Priniski, I Verma, F Morstatter, Pipeline for modeling causal beliefs from natural language in *Proceedings of the 61st Annual Meeting of the Association for Computational Linguistics (Volume 3: System Demonstrations).* (Association for Computational Linguistics, Toronto, Canada), pp. 436–443 (2023).
31. M Grootendorst, Bertopic: Neural topic modeling with a class-based TF-IDF procedure. *arXiv preprint arXiv:2203.05794* (2022).
32. Y Liu, Roberta: A robustly optimized BERT pretraining approach. *arXiv preprint arXiv:1907.11692* **364** (2019).
33. A Heiss, A guide to modeling outcomes that have lots of zeros with Bayesian hurdle lognormal and hurdle Gaussian regression models (2022).
